# Causal relationship between intervertebral disc degeneration and osteoporosis: a bidirectional two-sample Mendelian randomization study

**DOI:** 10.3389/fendo.2024.1298531

**Published:** 2024-04-30

**Authors:** Gaohua Liu, Hanjing Zhang, Meichun Chen, Wenkang Chen

**Affiliations:** ^1^ Institute of Clinical Medicine, The First Affiliated Hospital, Hengyang Medical School, University of South China, Hengyang, Hunan, China; ^2^ Department of Hepatobiliary Surgery, The First Affiliated Hospital, Hengyang Medical School, University of South China, Hengyang, Hunan, China; ^3^ Department of Hematology, Fujian Medical University Union Hospital, Fuzhou, Fujian, China; ^4^ Speciality of Sports Medicine in Department of Orthopaedics, The First Affiliated Hospital, Hengyang Medical School, University of South China, Hengyang, Hunan, China

**Keywords:** osteoporosis, bone mineral density, intervertebral disc degeneration, Mendelian randomization, genome-wide association studies

## Abstract

**Introduction:**

The relationship between intervertebral disc degeneration (IVDD) and osteoporosis (OP), diagnosed primarily using bone mineral density (BMD), remains unclear so far. The present study, therefore, aimed to investigate the potential relationship between osteoporosis and intervertebral disc degeneration using Mendelian randomization and genome-wide association analyses. Specifically, the impact of bone mineral density on the development of intervertebral disc degeneration was evaluated.

**Materials and methods:**

The genome-wide association studies (GWAS) summary data of OP/BMDs and IVDD were collected from the FinnGen consortium, the GEFOS consortium, and MRC-IEU. The relationship between IVDD and OP was then explored using TSMR. The inverse-variance weighted (IVW) method was adopted as the primary effect estimate, and the reliability and stability of the results were validated using various methods, including MR-Egger, weighted median, simple mode, weighted mode, and MR-PRESSO.

**Results:**

No significant causal relationship was observed between OP and IVDD (IVW, P > 0.05) or between femoral neck BMD (FA-BMD) and IVDD when OP and FA-BMD were used as exposures. However, increased levels of total body BMD (TB-BMD) and lumbar spine BMD (LS-BMD) were revealed as significant risk factors for IVDD (TB-BMD: IVW, OR = 1.201, 95% CI: 1.123–1.284, *P* = 8.72 × 10^−8^; LS-BMD: IVW, OR = 1.179, 95% CI: 1.083–1.284, *P* = 1.43 × 10^−4^). Interestingly, both heel BMD (eBMD) and femur neck BMD (FN-BMD) exhibited potential causal relationships (eBMD: IVW, OR = 1.068, 95% CI: 1.008–1.131, *P* = 0.0248; FN-BMD, IVW, OR = 1.161, 95% CI: 1.041–1.295, *P* = 0.0074) with the risk of IVDD. The reverse MR analysis revealed no statistically causal impact of IVDD on OP and the level of BMD (*P* > 0.05).

**Conclusion:**

OP and the level of FA-BMD were revealed to have no causal relationship with IVDD. The increased levels of TB-BMD and LS-BMD could promote the occurrence of IVDD. Both eBMD and FN-BMD have potential causal relationships with the risk of IVDD. No significant relationship exists between IVDD and the risk of OP. Further research is warranted to comprehensively comprehend the molecular mechanisms underlying the impact of OP and BMD on IVDD and vice versa.

## 1 Introduction

Intervertebral disc degeneration (IVDD) serves as the pathological basis for various spinal degenerative diseases that contribute to disability and reduce the quality of life of the affected patients (1). The intervertebral disc comprises the nucleus pulposus, the cartilage endplate, and the annulus fibrosus. Each of these three main components of the intervertebral disc is mostly composed of collagen and proteoglycan, both of which are crucial for imparting critical qualities to the disc (2). Intervertebral disc degeneration (IVDD) is a pathological condition characterized by a progressive decline in the levels of proteoglycans and the water content inside the nucleus pulposus. This degenerative condition is well-recognized as the primary contributor to the development of lower back pain (3). In severe cases, the destroyed discs compress the spinal nerve roots located at L4-S2, which often results in sciatica (4).

Osteoporosis, often referred to as OP, is a multifaceted skeletal disorder characterized by a loss in bone mass and impairment of bone microarchitecture, which results in heightened vulnerability to fractures and increased fragility of the bones (5). A key feature of osteoporosis is the reduction in bone mineral density (BMD). Dual-energy X-ray absorptiometry (DXA) is currently the primary method of establishing the clinical diagnosis of osteoporosis (6). OP is prevalent globally, affecting nearly 200 million individuals, particularly postmenopausal women (7). The important risk factors reported for OP include genetics, living habits, and medical history (5). The timely identification and management of osteoporosis (OP) is important as it would significantly contribute to preventing bone fractures and enhance the overall quality of life of the affected patients.

The correlation between osteoporosis and disc degeneration remains debatable in the scientific community to date. Liang et al. (8) demonstrated through a retrospective study that reduced vertebral bone mineral density (BMD), osteoporosis, and the exacerbation of disc degeneration were correlated. On the other hand, Kaiser et al. (9) demonstrated, through a prospective study, that higher trabecular BMD is negatively associated with disc height loss. Interestingly, certain retrospective and prospective studies have also reported no correlation between BMD and the progression of IVDD (10–13). These inconsistent conclusions could be originating from the confounders or covariates, which impact the association between BMD and IVDD.

IVDD and osteoporosis share a few genetic mechanisms and signaling pathways. Inflammatory cytokines, including interleukin-1 (IL-1), IL-6, and tumor necrosis factor-α (TNF-α), influence the development, progression, and severity of intervertebral disc degeneration (IVDD) (14–16) and OP (17–19). The matrix metallopeptidase 9 (MMP-9), which is an important proteolytic enzyme, is also closely associated with IVDD and OP. In addition, several same signaling pathways, including PI3K-AKT (20, 21), WNT/β-catenin (22, 23), and NF-κB (24, 25), are associated with intervertebral disc degeneration (IVDD) and also reported to significantly affect the pathogenesis of osteoporosis.

While numerous studies have reported the association between IVDD and OP, the direct causal relationship remains obscure. Mendelian randomization (MR) is a novel methodology that is being increasingly utilized to explore the causal associations between modifiable exposures and various illnesses or features (26, 27). MR allows for effectively overcoming certain limitations, especially unfeasible causality in the randomized controlled trials (RCT) and inevitable confounding bias or reverse causality in observational studies (26). MR also aids in distinguishing causal pathways from risk factors that are challenging to randomize or prone to measurement errors (28).

Our study indicates no discernible causal association between OP and the risk of intervertebral disc degeneration (IVDD) or between FA-BMD and the risk of IVDD. The elevated levels of TB-BMD and LS-BMD could, however, contribute to the development of intervertebral disc degeneration (IVDD). In addition, both eBMD and FN-BMD were revealed to have a putative causal relationship with the risk of intervertebral disc degeneration (IVDD). Notably, no substantial correlation was revealed between intervertebral disc degeneration (IVDD) and osteoporosis (OP) or among the bone mineral density across various anatomical locations and different age cohorts.

## 2 Methods

### 2.1 Data resources and sample information

**Figure 1:**
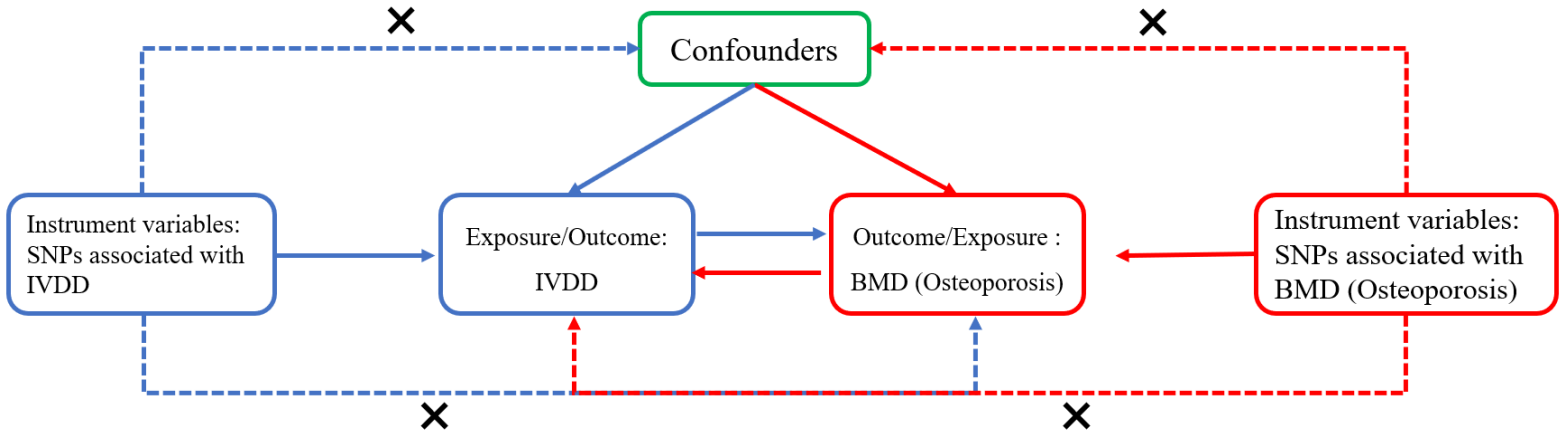
The flowchart for the two-sample bidirectional Mendelian randomization analysis. The blue line represents the Mendelian randomization analysis of the causal relationship of intervertebral disc degeneration (IVDD) with bone mineral density (BMD) or osteoporosis. The red line represents the Mendelian randomization analysis of the causal relationship of bone mineral density or osteoporosis with IVDD. The following three assumptions had to be considered: the selected instrument variants (SNPs) should be significantly associated with the exposure; the variants should be independent of any exposure–outcome relationship confounders; the variants affect the outcome only via the exposure.

The study design is depicted in **Figure 1**. The study was conducted by following the STROBE-MR guidelines (29). The data used in the present study were the summary genome-wide association studies (GWAS) data of intervertebral disc degeneration (IVDD) and osteoporosis, which were obtained from the FinnGen collaboration. The IVDD dataset comprised 20,001 cases and 164,692 controls, whereas the osteoporosis dataset comprised 3,203 cases and 209,575 controls (30). The genome-wide association study (GWAS) data on lumbar spine bone mineral density (LS-BMD), femur neck bone mineral density (FN-BMD), and forearm bone mineral density (FA-BMD) collected by the Genetic Factors for Osteoporosis (GEFOS) consortium were also used. The dataset comprised 25,509 cases for FN-BMD, 12,906 cases for LS-BMD, and 563 cases for FA-BMD (31, 32). In addition, heel bone mineral density (eBMD) data were obtained from the MRC-IEU, and the dataset comprised 265,627 individuals. The data for TB-BMD for different age groups were obtained from the reports of a large GWAS meta-analysis study (32). The TB-BMD, LS-BMD, FN-BMD, and FA-BMD assessments were conducted using dual-energy X-ray absorptiometry (DXA) (6). The eBMD assessments were conducted using ultrasonography (33). A reverse analysis was conducted to obtain the genetic instruments for OP and bone mineral density (BMD) at different anatomical sites, and their impact on intervertebral disc degeneration (IVDD) was determined. IVDD was diagnosed using the International Classification of Diseases 10^th^ Revision (ICD-10) M51, ICD-9 722, and ICD-8 275, with removed ICD-9 7220|7224|7227|7228Av and ICD-8 7250. Detailed information of these GWAS summary data is provided in **Supplementary Table 1**.

### 2.2 MR analysis

#### 2.2.1 Selection of genetic variants

In order to address the issue of weak instrument bias, a genome-wide significance threshold of P < 5 × 10^−8^ was used as the default criterion for identifying the single-nucleotide polymorphisms (SNPs), whereas the threshold of minor allele frequency (MAF) > 0.01 was used as the genetic instruments for osteoporosis (OP), bone mineral density (BMD), and intervertebral disc degeneration (IVDD). Next, the clumping threshold (the genetic distance = 10,000 kb and the SNP linkage disequilibrium value r^2^ < 0.001) was established using the data from the 1000 genomes project for European ancestry (34) to resolve the linkage disequilibrium (LD) issue based on the identified SNPs.

Diabetes, BMI, and smoking are significant prognostic factors for both OP and IVDD. Several scholars have proposed that the risk factors for diabetes mellitus (35, 36) and body mass index (BMI) are associated with IVDD (37). Moreover, a retrospective study indicated that cigarette smoking accelerates the development of cervical disc degeneration (38). Interestingly, a higher level of education was reported as a risk factor for cervical disc degeneration, regardless of age differences among the respondents (39). OP is also reported to be closely associated with diabetes mellitus, BMI (40), smoking (41, 42), and the level of education (43). BMI and smoking are considered to have significant effects on OP, particularly in postmenopausal women. Accordingly, to ensure obtaining statistically meaningful outcomes, the present study used *PhenoScannerV2* to identify and exclude certain confounding single-nucleotide polymorphisms (SNPs) associated with diabetes, body mass index (BMI), smoking, and social status in our study.

The strength of the IVs was assessed by computing the F-statistics using the formula F = R^2^ × (N – 2)/(1 – R^2^). In the formula, R^2^ denotes the proportion of variation in the exposure variable explained by the IV, and N denotes the sample size of the original GWAS used as the outcome variable (44). The R^2^ for each IV was computed using the formula R^2^ = [2 × EAF × (1 – EAF) × beta^2^)/[(2 × EAF × (1 – EAF) × beta^2^] + [2 × EAF × (1 – EAF) × N × (SE × beta^2^)]. In this formula, EAF denotes the effect of allele frequency, beta represents the estimated genetic effect on the outcome, N denotes the sample size of the genome-wide association study (GWAS), and SE denotes the standard error of the genetic effect (45). The IVs with the F statistics over 10 were considered credible instrumental variables and selected for the subsequent Mendelian randomization study.

#### 2.2.2 Sensitivity analysis and statistical analysis

The potential causal relationship between intervertebral disc abnormalities and osteoporosis was investigated in the present study primarily using the inverse variance weighted (IVW) method, as described in a previous report (46). The Cochrane’s Q test was conducted to evaluate the heterogeneity among the single-nucleotide polymorphisms (SNPs). Furthermore, the strength and reliability of the primary results obtained in the previous steps were assessed using various sensitivity analyses, including the weighted-median approach and the IVW method. Moreover, the possible presence of directional pleiotropy was evaluated using MR-Egger regression (47). The slope of MR-Egger regression indicates the causal estimates that have been adjusted for pleiotropy, whereas the intercept’s value provides an estimation of the extent of pleiotropy. In addition, the Mendelian randomization pleiotropy residual sum and outlier (MR-PRESSO) test was conducted as a supplementary approach to identify and address the horizontal pleiotropic outliers (48). A global test of heterogeneity was conducted through a regression analysis of the relationships between the single-nucleotide polymorphisms (SNPs) and the outcomes while considering the connections between the SNPs and the exposures. The observed distance of each SNP from the regression was then compared with the anticipated distance according to the null hypothesis of no pleiotropy. The robustness of the obtained results was assessed using “leave-one-out” analyses, which involved systematically eliminating one single SNP at a time. This was followed by a reanalysis of the Mendelian randomization (MR) results using the IVW approach with the remaining instrumental variables (IVs).

### 2.3 Calculation of statistical power

The statistical power was evaluated using the mRnd website (https://shiny.cnsgenomics.com/mRnd/) (49). The key determinants of statistical power were the size of the sample for the result and the extent to which the genetic instrument accounted for the variance in the exposure variable.

All analyses were performed using R (version 4.1.2) and the related R packages (TwoSampleMR and forestplot). A *P*-value of <0.005 (0.05/10) indicated strong evidence of a causal association after the Bonferroni correction threshold was applied. A *P*-value ranging between 0.05 and 0.005 was considered suggestive evidence for a potential causal association.

## 3 Results

### 3.1 Causal effects of osteoporosis and bone mineral density on IVDD

All the IVs that were ultimately selected exhibited F-statistic values of over 10. Details of the method adopted to screen out the single-nucleotide polymorphisms (SNPs) are provided in **Supplementary Table 2**. In the analysis of the effect of eBMD on the risk of IVDD, rs7816131 was excluded because of its robust association with IVDD (*P* = 8.3 × 10^−9^), which is not in accordance with the third assumption of MR analysis. Finally, 2 SNPs of OP, 80 SNPs of TB-BMD, 22 SNPs of LS-BMD, 20 SNPs of FN-BMD, 3 SNPs of FA-BMD, and 331 SNPs of eBMD were used as IVs to analyze the relationship between OP/BMDs and the risk of intervertebral disc degeneration (IVDD). Comprehensive data on the IVs for BMD are provided in **Supplementary Table 3**. The variation explained by these IVs was 0.02% for OP, 9.05% for TB-BMD, 2.1% for FN-BMD, 1.65% for FA-BMD, 2.27% for LS-BMD, and 14.27% for eBMD. The statistical power values determined in the Mendelian randomization analyses of OP and BMD in terms of their effects on IVDD are presented in **Supplementary Table 5**.

**Figure 2:**
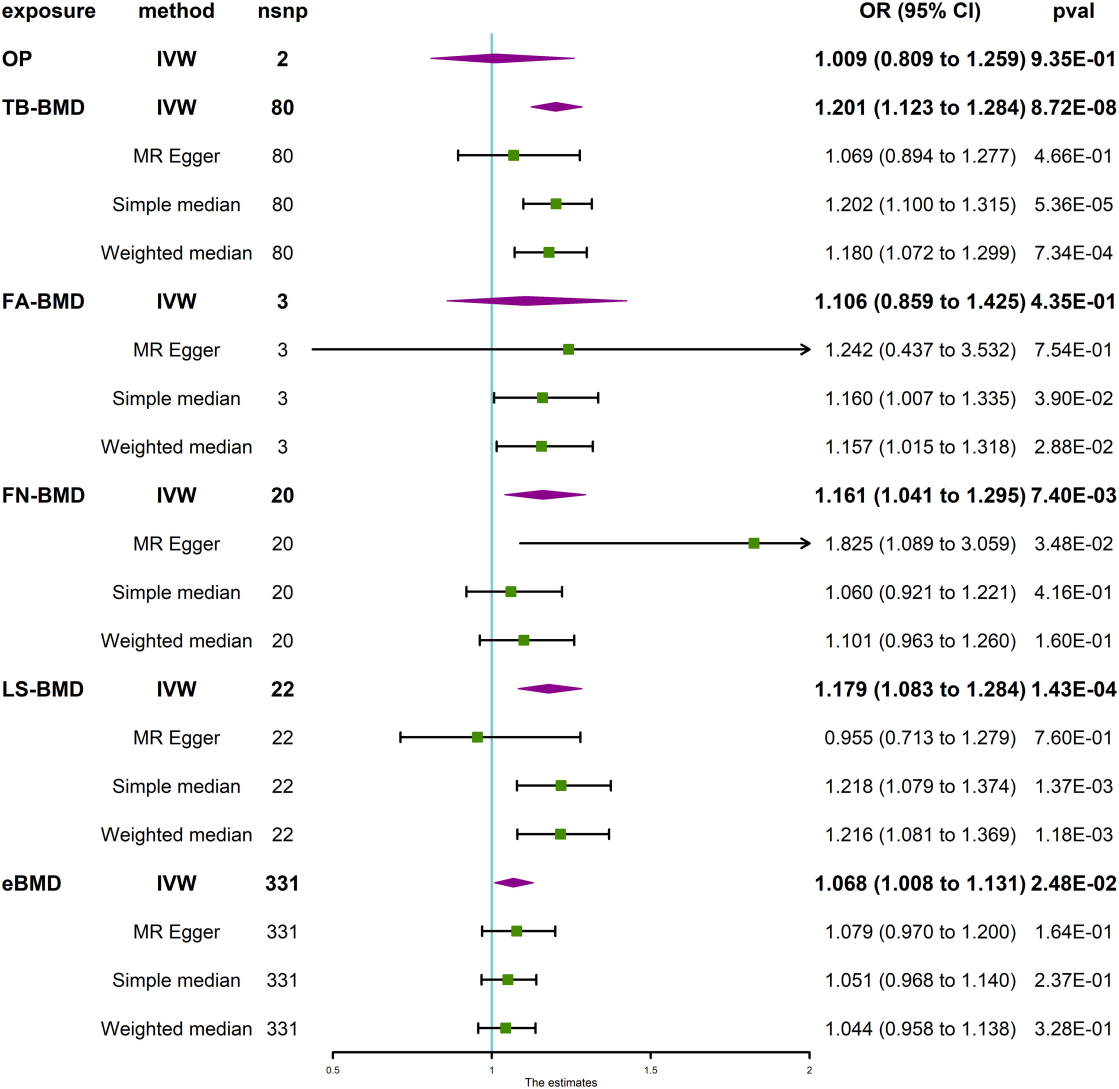
Causal effect of OP and BMD at different anatomical sites on IVDD. IVDD, intervertebral disc degeneration; OP, osteoporosis; TB-BMD, total body bone mineral density; FN-BMD, femoral neck bone mineral density; FA-BMD, forearm bone mineral density; LS-BMD, lumbar spine bone mineral density; eBMD, heel bone mineral density; IVW, inverse variance weighted; nsnp, number of single nucleotide polymorphisms; CI, confidence interval.

The MR analysis did not yield statistically significant evidence of the causal effects of OP (IVW, *P* > 0.05) and FA-BMD (IVW, *P* > 0.05) on IVDD (**Figure 2**). However, positive correlations of the BMDs (TB-BMD, LS-BMD, and eBMD) with the risk of IVDD were revealed. The results indicated a significant association between the genetically enhanced BMD levels and a higher susceptibility to IVDD (TB-BMD: IVW, OR = 1.201, 95% CI: 1.123–1.284, *P* = 8.72 × 10^−8^; LS-BMD: IVW, OR = 1.179, 95% CI: 1.083–1.284, *P* = 1.43 × 10^−4^; **Figure 2**). The different results may be attributed to too few amounts of SNPs associated with OP and FA-BMD which served as exposures. Thus, the results of the MR-Egger and (or) MR-PRESSO test are not available. Moreover, heterogeneity (P for Cochrane’s Q in IVW = 0.049 or 0.005 when OP and FA-BMD were served as exposures, **Table 1**) may exert an influence on this difference (**Table 1**).

**Table 1:** MR sensitivity analyses of IVDD and BMD at different sites and in different age groups.

Exposure	Outcome	Inverse variance weighted			MR-Egger			MR-PRESSO Global Test
		Cochran Q	Q_df	Q_Pval	Intercept	Se	P value	P value
**OP**	**IVDD**	3.9	1	0.049	NA	NA	NA	NA
**TB-BMD**	**IVDD**	113.4	79	0.007	0.007	0.005	0.171	0.011
**FA-BMD**	**IVDD**	10.5	2	0.005	−0.016	0.068	0.856	NA
**FN-BMD**	**IVDD**	26.5	19	0.118	−0.029	0.016	0.097	0.097
**LS-BMD**	**IVDD**	22.9	21	0.349	0.016	0.011	0.156	0.37
**eBMD**	**IVDD**	515.2	330	<0.0001	−0.0004	0.002	0.829	<0.001
**IVDD**	**OP**	6.6	5	0.250	0.038	0.119	0.764	0.277
**IVDD**	**FN-BMD**	3.4	5	0.639	0.021	0.027	0.486	0.67
**IVDD**	**FA-BMD**	2.9	5	0.711	0.049	0.056	0.434	0.747
**IVDD**	**eBMD**	3.2	4	0.53	−0.001	0.011	0.931	0.545
**IVDD**	**LS-BMD**	7.3	5	0.198	−0.002	0.043	0.973	0.239
**IVDD**	**TB-BMD**	11.5	5	0.042	0.054	0.021	0.064	0.076
**IVDD**	**TB-BMD** **(age 0−15)**	11.9	5	0.036	−0.022	0.076	0.782	0.061
**IVDD**	**TB-BMD** **(age 15–30)**	8.1	5	0.150	0.193	0.080	0.074	0.177
**IVDD**	**TB-BMD** **(age 30–45)**	2.2	5	0.815	0.049	0.052	0.397	0.814
**IVDD**	**TB-BMD** **(age 45–60)**	3.1	5	0.692	0.012	0.038	0.776	0.657
**IVDD**	**TB-BMD (age over 60)**	9.7	5	0.084	0.084	0.035	0.075	0.11

IVDD, intervertebral disc degeneration; OP, osteoporosis; TB-BMD, total body bone mineral density; FN-BMD, femoral neck bone mineral density; FA-BMD, forearm bone mineral density; LS-BMD, lumbar spine bone mineral density; eBMD, heel bone mineral density; NA, not available.

MR sensitivity analysis of TB-BMD and IVDD exerted heterogeneous (P for Cochrane’s Q in IVW = 0.007, **Table 1**) and no horizontal pleiotropy (P for MR-Egger intercept = 0.171, **Table 1**) when TB-BMD was served as the exposure. The MR-Egger analysis exhibited less statistical power, as seen by the lack of a significant P value and broader confidence intervals when compared with the IVW technique (50). Therefore, the MR-PRESSO Outlier Test, another horizontal pleiotropy test, was performed. The SNP rs4846580 was identified as an outlier using the MR-PRESSO Outlier Test, with an observed residual sum of squares (RSSobs) value of 1.92 × 10^−3^ (*P* < 0.08). Therefore, this SNP was removed during the analysis of the relationship between TB-BMD and the risk of IVDD. The results of the MR analysis indicated no statistically significant association between TB-BMD and IVDD (IVW, OR = 1.073, 95% CI: 1.014–1.135, *P* = 1.47 × 10^−2^) when the analysis was repeated with the remaining five single-nucleotide polymorphisms (SNPs) (**Supplementary Figure 5**). Notably, neither heterogeneity nor horizontal pleiotropy was indicated (P for Cochrane’s Q in IVW = 0.349; P for MR-Egger intercept = 0.016, P for MR-PRESSO Global Test = 0.37, **Table 1**) in the MR sensitivity analysis of LS-BMD with IVDD.

Interestingly, both eBMD and FN-BMD were revealed to have potential causal relationships (eBMD: IVW, OR = 1.068, 95% CI: 1.008–1.131, *P* = 0.0248; FN-BMD, IVW, OR = 1.161, 95% CI: 1.041–1.295, *P* = 7.40 × 10^−3^; **Figure 2**) with the risk of IVDD according to statistical significance standard (P value threshold) defined in the Method section. Neither heterogeneity nor horizontal pleiotropy was indicated in the directional pleiotropy in the MR sensitivity analysis of FN-BMD with IVDD (P for Cochran’s Q in IVW = 0.118, MR-Egger intercept = −0.029, P = 0.097; P for MR-PRESSO Global Test = 0.37, **Table 1**). However, horizontal pleiotropy and heterogeneity (P for MR-PRESSO Global Test <0.001, P for Cochrane’s Q in IVW <0.0001, **Table 1**) were indicated in the MR sensitivity analysis of eBMD with IVDD, although P for the MR-Egger intercept is more than 0.05 (**Table 1**). This may be attributed to too many amounts of SNPs associated with eBMD that served as the exposures. The scatter plots for the effect sizes of the SNPs for OP and TB-BMD at different anatomical sites on IVDD are presented in **Supplementary Figure 1**. The forest plots, leave-one-out analysis plots, and funnel plots for the causal effect of BMD on IVDD are depicted in **Supplementary Figures 2-4**. When the leave-one-out analysis was conducted using the IVW method, most of the determined correlations remained unchanged even when considering a single SNP associated with bone mineral density (BMD).

### 3.2 Causal effect of IVDD on BMD at different anatomical sites or in different age groups

Six SNPs (rs3010043, rs4473430, rs3135840, rs6470763, rs4284332, and rs17487277) were selected as IVs for determining the causal effect of IVDD on the risk of OP, TB-BMD, FN-BMD, FA-BMD, and LS-BMD. Notably, rs6470763 was directly associated with heel BMD (beta = 0.021, *P* = 7.60 × 10^−11^), which is not in accordance with the third assumption of MR analysis. Therefore, this SNP was removed from the analysis of the relationship between intervertebral disc degeneration (IVDD) and the risk of eBMD. All IVs that were finally selected for the analysis had F-statistic values over 10. Detailed information on the IVs strongly associated with IVDD is provided in **Supplementary Table 4**. These IVs accounted for a variance of 0.11% or 1.20% in the analysis of the effect of IVDD on BMD.

**Figure 3:**
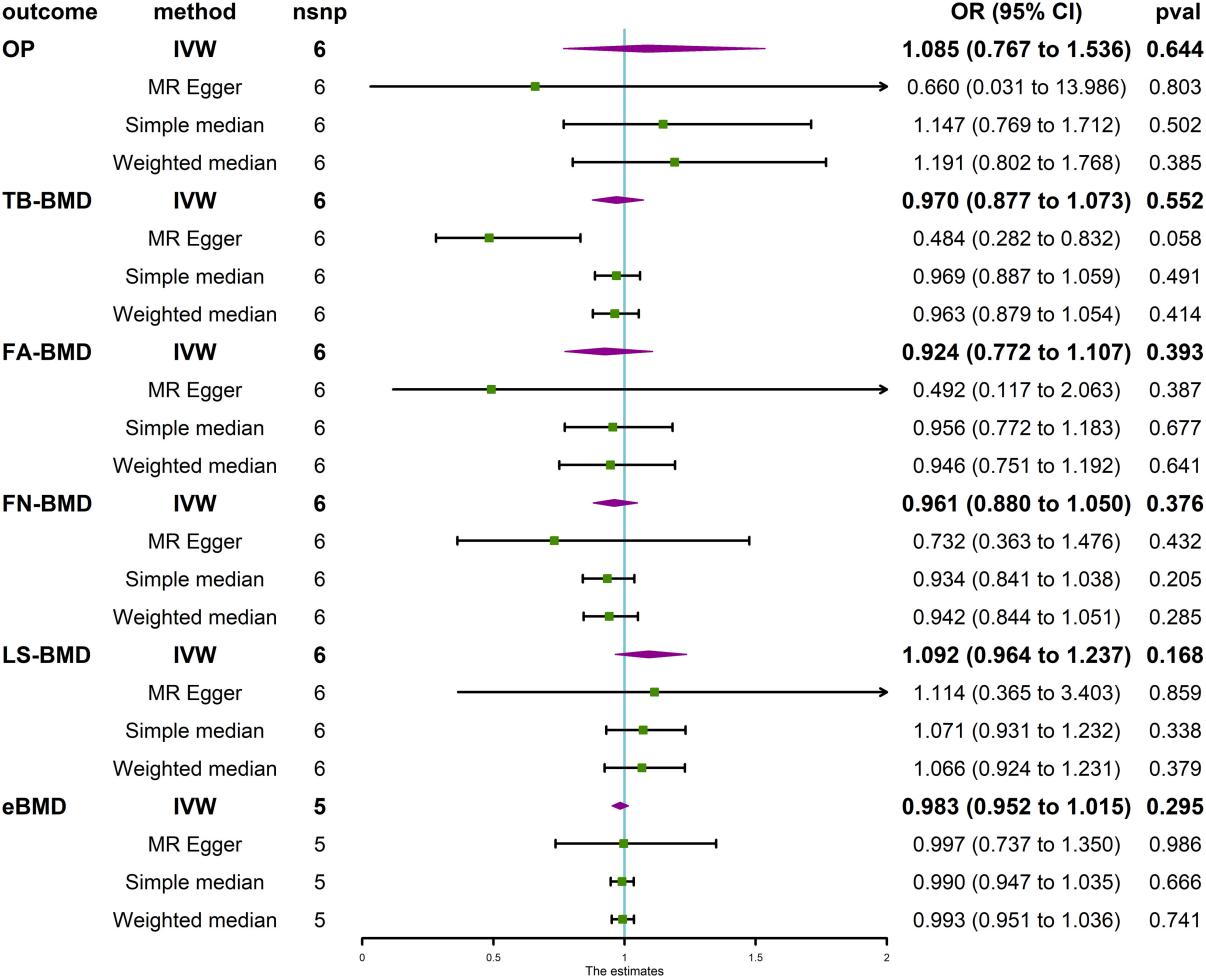
Causal effect of IVDD on OP and BMD at different anatomical sites. IVDD, intervertebral disc degeneration; OP, osteoporosis; TB-BMD, total body bone mineral density; FN-BMD, femoral neck bone mineral density; FA-BMD, forearm bone mineral density; LS-BMD, lumbar spine bone mineral density; eBMD, heel bone mineral density; IVW, inverse variance weighted; nsnp, number of single-nucleotide polymorphisms; CI, confidence interval.

**Figure 4:**
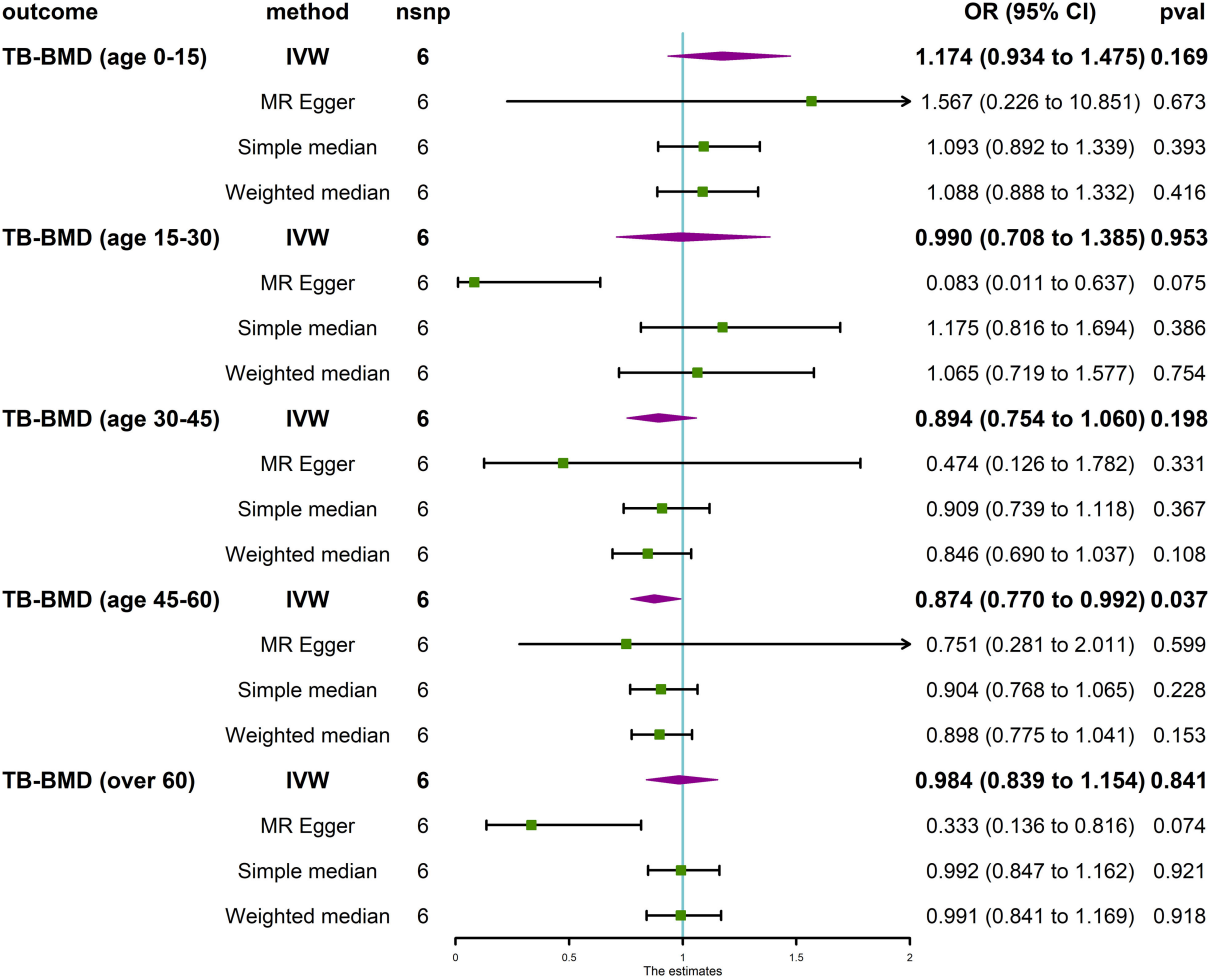
Causal effect of IVDD on BMD in different age groups. TB-BMD, total body bone mineral density; IVW, inverse variance weighted; nsnp, number of single-nucleotide polymorphisms; OR, odds ratio; CI, confidence interval.

The MR analysis revealed that IVDD was not associated with BMD at different anatomical sites (**Figure 3**) and in different age groups (**Figure 4**). Neither heterogeneity nor horizontal pleiotropy (all P for Cochran’s Q in IVW, MR-Egger intercept, and MR-PRESSO Global Test are more than 0.05) was indicated in the MR sensitivity analysis of IVDD with BMD at different sites, and in different age groups, excluded heterogeneity was found in the analysis of IVDD with TB-BMD and TB-BMD (age 0–15) (P for Cochrane’s Q in IVW is less than 0.05). Otherwise, the scatter plots for the effect sizes of SNPs for IVDD’s relationship to OP and TB-BMD at different anatomical sites are depicted in **Supplementary Figures 6, 7**. The forest plots, leave-one-out analysis plots, and funnel plots for the causal effect of IVDD on BMDs are depicted in **Supplementary Figures 8, 9**.

## 4 Discussion

The present study revealed no significant evidence to support the causal effect of OP and FA-BMD on the risk of IVDD. Moreover, the positive relationships of TB-BMD and LS-BMD with the risk of IVDD were revealed. In addition, both eBMD and FN-BMD exhibited potential causal relationships with the risk of IVDD. In reverse MR analysis, no causal effects of IVDD on the risk of OP and the change in bone mineral density were revealed.

The scientific community continues to debate whether bone mineral density (BMD) affects IVDD and, if so, how. While certain studies have reported osteoporosis as a causal factor of IVDD, others have considered it a protective factor against IVDD. Otherwise, several studies demonstrate there is no association between OP and IVDD (12, 51).

It has long been suggested that diminished bone quality is associated with the gradual deterioration of the endplates and the development of spondylosis, which ultimately results in heightened disc degeneration (52). Nevertheless, the incidence of intervertebral disc degeneration (IVDD) is lower among individuals with a low bone mineral density (BMD), even though these people are more susceptible to vertebral body fractures (53–55). According to this concept, it is postulated that osteoporosis, a degenerative and incapacitating disorder associated with aging and affecting a significant population globally (56), potentially delays the onset of intervertebral disc degeneration (IVDD). Functional investigations aimed at elucidating the correlation between the genetic factors influencing bone mineral density (BMD) and intervertebral disc degeneration (IVDD) are expected to provide valuable insights into this association and facilitate the discovery of novel treatment approaches.

Mechanistically, some scholars believe that osteoporosis leads to lower vertebral BMD, aggravates disc load, reduces the supply of nutrients to the intervertebral disc (57), and increases inflammatory factors that cause disc degeneration (15, 58). Others consider osteoporosis to delay disc degeneration that the vertebral body with lower BMD has a loose bony microstructure, which allows the vascular buds that nourish the cartilage endplate to grow better, and the increase in the number of vascular buds to enrich the blood supply of the cartilage endplate and can better provide nutrients to the intervertebral discs, thereby delaying IVDD (59, 60). In terms of genetic predictions, our results support the latter opinion that increased levels of TB-BMD and LS-BMD are the risk factors for IVDD, whereas eBMD and FN-BMD were revealed as the potential risk factors for IVDD. On the other hand, the results of the present study revealed no causal effect of OP on IVDD when just two SNPs were selected as IVs. These different results could be attributed to the different number of SNPs associated with the exposure and types of exposure variables. Furthermore, no statistically significant correlation was observed between intervertebral disc degeneration (IVDD) and osteoporosis (OP) or among the bone mineral density at different anatomical locations and in different age groups.

The present MR study offers several advantages. The use of Mendelian randomization effectively mitigated the potential influences of confounding bias and reverse causation. In addition, several sensitivity analyses were conducted to assess the robustness of the three Mendelian randomization assumptions, which reduced the likelihood of spurious findings. The instrumental variable weighted (IVW) method, which was adopted as the principal approach in the present study, resulted in a superior statistical power compared with other Mendelian randomization (MR) approaches, particularly the MR-Egger approach (50). Consequently, the MR-Egger analysis led to lower statistical power, as evident in the lack of a significant P value and broader confidence intervals compared with the IVW technique. The findings of the present study provided additional support for the necessity of maintaining a constant beta direction across all magnetic resonance (MR) procedures. Furthermore, harmonized data were consistently identified and rectified using the MR-PRESSO method (48). This ensured the absence of horizontal pleiotropy throughout the Mendelian randomization (MR) analysis and enhanced the reliability of the obtained findings.

Nonetheless, similar to other studies, the present study also had certain limitations. The first limitation is that the study participants were exclusively of European origin, which may cause the findings of this study not to be generalizable to individuals of other nations, such as those of African or East Asian descent. Furthermore, the comprehensive elimination of pleiotropy was challenging due to the limited understanding of the overall biological functionality of these instrumental variations. In addition, while the findings of the present study indicated the existence of potential causal relationships between bone mineral density at various anatomical sites and the risk of intervertebral disc degeneration (IVDD), a further comprehensive investigation of the intricate underlying processes is nonetheless warranted. Lastly, it is difficult to validate the findings of the present study in wet lab assays.

In summary, the present study revealed no substantial causal relationship, either directly from the causative impact of OP or indirectly via FA-BMD, on IVDD. The elevation in the levels of TB-BMD (total body bone mineral density) and LS-BMD (lumbar spine bone mineral density) could, however, contribute to the development of intervertebral disc degeneration (IVDD). Both eBMD and FN-BMD exhibited a putative causal relationship with the risk of intervertebral disc degeneration (IVDD). Furthermore, the association of intervertebral disc degeneration (IVDD) with osteoporosis (OP) and bone mineral density (BMD) was not statistically significant. Therefore, further investigation is required to elucidate the molecular mechanisms underlying the impact of bone mineral density (BMD) on intervertebral disc degeneration (IVDD), which will facilitate the precise treatment of these two bone degenerative diseases by orthopedic surgeons.

## Data availability statement

The datasets presented in this study can be found in online repositories. The names of the repository/repositories and accession number(s) can be found in the article/**Supplementary material**.

## Author contributions

GL: Investigation, Validation, Writing – review & editing, Conceptualization, Data curation, Formal analysis, Writing – original draft, Visualization, Software, Project administration, Methodology. HZ: Writing – review & editing, Supervision, Resources, Funding acquisition, Conceptualization. MC: Writing – original draft, Visualization, Validation, Methodology, Formal analysis, Data curation, Conceptualization. WC: Funding acquisition, Supervision, Writing – review & editing, Investigation, Validation.
