# Supplementary material for: Causal relationship between intervertebral disc degeneration and osteoporosis: a bidirectional two-sample Mendelian randomization study
Source: Front Endocrinol (Lausanne). 2024 Apr 30;15:1298531. doi: 10.3389/fendo.2024.1298531 (PMC11091238; doi:10.3389/fendo.2024.1298531)
Supplement: Supplementary file 1 [file DataSheet_1.docx]

**Supplementary materials**

**Tables**

Supplementary Table 1. The detailed data information of the Mendelian randomization study on the association of IVDD with the risk of OP.

Supplementary Table 2. Details of the BMD-associated proxy SNPs in the Mendelian randomization analyses of BMD at different sites on IVDD

Supplementary Table 3. Instrument variants for the Mendelian randomization analyses of OP and BMD on IVDD

Supplementary Table 4. Instrument variants for the Mendelian randomization analyses of IVDD on OP and BMD

Supplementary Table 5. Statistical power for the Mendelian randomization analyses of BMD on IVDD

**Figures**

Supplementary Figure 1. Scatter plot of the causal effect of of OP and IVDD using inverse-variance weighted, simple median, MR-Egger, and weighted median.

Supplementary Figure 2. Forest plot for causal effect of OP (A), TB-BMD (B), FN-BMD (C), FA-BMD (D), LS-BMD (E), eBMD (F) on IVDD.

Supplementary Figure 3. Leave-one-out plot for causal effect of TB-BMD (A), FN-BMD (B), FA-BMD (C), LS-BMD (D), eBMD (E) on IVDD.

Supplementary Figure 4. Funnel plot for causal effect of OP (A), TB-BMD (B), FN-BMD (C), FA-BMD (D), LS-BMD (E), and eBMD (F) on IVDD.

Supplementary Figure 5. Mendelian randomization analysis results for the effects of TB-BMD on IVDD after removal of potentially pleiotropic SNP (rs4846580)

Supplementary Figure 6. Scatter plot of the causal effect of IVDD and BMD at different sites using inverse-variance weighted, simple median, MR-Egger, and weighted median.

Supplementary Figure 7. Scatter plot of the causal effect of IVDD and BMD in different age groups using inverse-variance weighted, simple median, MR-Egger, and weighted median.

Supplementary Figure 8. MR sensitivity analysis of IVDD on BMD at different ages.

Supplementary Figure 9. MR sensitivity analysis of IVDD on BMD at different sites

Supplementary Table 1. The detailed data information of the Mendelian randomization study on the association of IVDD with the risk of OP.

| **Exposures**  **or outcome** | **Sample size  (total or cases/controls)** | **Ancestry** | **Consortia** | **PMID** | **URL of available datasets** |
| --- | --- | --- | --- | --- | --- |
| **Intervertebral disc degeneration** | 20,001/164,692 | European | The FinnGen consortium | NA | <https://gwas.mrcieu.ac.uk/datasets/>finn-b-M13_INTERVERTEB/ |
| **Osteoporosis** | 3203/209,575 | European | The FinnGen consortium | NA | <https://gwas.mrcieu.ac.uk/datasets/>finn-b-M13_OSTEOPOROSIS/ |
| **Femoral neck**  **bone mineral density** | 32,735 | Mixed | The GEFOS Consortium | 26367794 | <https://gwas.mrcieu.ac.uk/datasets/>ieu-a-980/ |
| **Forearm bone mineral density** | 8143 | Mixed | The GEFOS Consortium | 26367794 | <https://gwas.mrcieu.ac.uk/datasets/>ieu-a-977/ |
| **Lumbar spine**  **bone mineral density** | 28,498 | Mixed | The GEFOS Consortium | 26367794 | <https://gwas.mrcieu.ac.uk/datasets/>ieu-a-982/ |
| **Heel bone mineral density** | 265,627 | European | MRC-IEU | NA | <https://gwas.mrcieu.ac.uk/datasets/>ukb-b-8875/ |
| **Total body bone mineral density** | 56,284 | European | GWAS meta-analysis study | 29304378 | <https://gwas.mrcieu.ac.uk/datasets/>  ebi-a-GCST005348/ |
| **Total body bone mineral density (age 0-15)** | 11,807 | Mixed (above  86% European) | GWAS meta-analysis study | 29304378 | <https://gwas.mrcieu.ac.uk/datasets/>  ebi-a-GCST005345/ |
| **Total body bone mineral density (age 15-30)** | 4180 | Mixed (above  86% European) | GWAS meta-analysis study | 29304378 | <https://gwas.mrcieu.ac.uk/datasets/>  ebi-a-GCST005344/ |
| **Total body bone mineral density (age 30-45)** | 10,062 | Mixed (above  86% European) | GWAS meta-analysis study | 29304378 | <https://gwas.mrcieu.ac.uk/datasets/>  ebi-a-GCST005346/ |
| **Total body bone mineral density (age 45-60)** | 18,805 | European | GWAS meta-analysis study | 29304378 | <https://gwas.mrcieu.ac.uk/datasets/>  ebi-a-GCST005350/ |
| **Total body bone mineral density (age over 60)** | 22,504 | Mixed (above  86% European) | GWAS meta-analysis study | 29304378 | <https://gwas.mrcieu.ac.uk/datasets/>  ebi-a-GCST005349/ |

Supplementary Table 2. Details of the BMD-associated proxy SNP in the Mendelian randomization analyses of BMD at different sites on IVDD

| **SNP** | **TB-BMD** | **eBMD** | **FN-BMD** | **LS-BMD** |
| --- | --- | --- | --- | --- |
| **rs838721** | rs7584554 |  |  |  |
| **rs10992875** |  | rs12551601 |  |  |
| **rs113869252** |  | rs1406225 |  |  |
| **rs11576308** |  | rs993471 |  |  |
| **rs12443252** |  | rs3862433 |  |  |
| **rs62038775** |  | rs80454 21 |  |  |
| **rs7072196** |  | rs10795055 |  |  |
| **rs77847666** |  | rs75471337 |  |  |
| **rs79854277** |  | rs79950712 |  |  |
| **rs13194508** |  |  | rs55901622 |  |
| **rs4281029** |  |  | rs4623318 |  |
| **rs9749364** |  |  |  | rs12459751 |

Supplementary Table 3. Instrument variants for the Mendelian randomization analyses of OP or BMD on IVDD

| **SNP** | **Chr** | **Pos** | | **EA/OA** | | **Beta** | **SE** | | **MAF** | | ***P*** | | **F** | **R^2^** | **Sample Size** |
| --- | --- | --- | --- | --- | --- | --- | --- | --- | --- | --- | --- | --- | --- | --- | --- |
| **Genetic variants of Osteoporosis** | | |  |  |  |  |  |  |  |  |  |  |  |  |  |
| rs577348 | 9 | 124330647 | | G/T | | 0.1568 | 0.0287 | | 0.2909 | | 4.47E-08 | | 29.8486 | 0.0001 | 212778 |
| rs61638232 | 16 | 92288 | | C/G | | 0.2259 | 0.041 | | 0.1228 | | 3.69E-08 | | 30.3571 | 0.0001 | 212778 |
| **Genetic variants of Total body bone mineral density** | | | | |  |  |  |  |  |  |  |  |  |  |  |
| rs10048745 | 2 | 68962137 | | A/G | | -0.0389 | 0.0067 | | 0.2477 | | 6.44E-09 | | 33.7081 | 0.0006 | 56284 |
| rs1037011 | 12 | 107302778 | | C/T | | 0.0404 | 0.0057 | | 0.5208 | | 1.54E-12 | | 50.234 | 0.0009 | 56284 |
| rs10490046 | 2 | 40630678 | | C/A | | -0.0429 | 0.0067 | | 0.2391 | | 1.43E-10 | | 40.9968 | 0.0007 | 56284 |
| rs10493013 | 1 | 22703035 | | C/T | | 0.1013 | 0.0074 | | 0.1811 | | 4.07E-43 | | 187.3872 | 0.0033 | 56284 |
| rs10735851 | 12 | 53743064 | | A/G | | -0.0541 | 0.0063 | | 0.7083 | | 5.84E-18 | | 73.7391 | 0.0013 | 56284 |
| rs10777212 | 12 | 90334829 | | T/G | | 0.0452 | 0.006 | | 0.3455 | | 5.05E-14 | | 56.7491 | 0.001 | 56284 |
| rs10788264 | 10 | 124015986 | | A/G | | -0.0338 | 0.0057 | | 0.4815 | | 2.61E-09 | | 35.1616 | 0.0006 | 56284 |
| rs10832520 | 11 | 15816918 | | A/T | | 0.1123 | 0.0158 | | 0.0394 | | 1.00E-12 | | 50.5161 | 0.0009 | 56284 |
| rs10901216 | 9 | 133471891 | | A/G | | -0.0474 | 0.0061 | | 0.3438 | | 5.53E-15 | | 60.3784 | 0.0011 | 56284 |
| rs10931982 | 2 | 202832130 | | C/T | | 0.0508 | 0.009 | | 0.7903 | | 1.59E-08 | | 31.8586 | 0.0006 | 56284 |
| rs11228240 | 11 | 68218290 | | T/C | | -0.083 | 0.0067 | | 0.2574 | | 1.72E-35 | | 153.4586 | 0.0027 | 56284 |
| rs1159798 | 10 | 54412493 | | C/A | | -0.0429 | 0.007 | | 0.7598 | | 1.01E-09 | | 37.5581 | 0.0007 | 56284 |
| rs11745493 | 5 | 122847622 | | G/A | | -0.0445 | 0.0065 | | 0.2537 | | 7.74E-12 | | 46.8682 | 0.0008 | 56284 |
| rs117557198 | 12 | 49655948 | | G/A | | 0.0769 | 0.012 | | 0.0676 | | 1.58E-10 | | 41.0653 | 0.0007 | 56284 |
| rs118115924 | 12 | 49379537 | | T/G | | -0.2822 | 0.0301 | | 0.0139 | | 6.99E-21 | | 87.8953 | 0.0016 | 56284 |
| rs11898505 | 2 | 54684557 | | G/A | | -0.0342 | 0.006 | | 0.6674 | | 1.28E-08 | | 32.4888 | 0.0006 | 56284 |
| rs11904127 | 2 | 85484818 | | A/G | | -0.0324 | 0.0057 | | 0.5512 | | 1.18E-08 | | 32.3091 | 0.0006 | 56284 |
| rs11910328 | 21 | 40350744 | | A/G | | -0.0429 | 0.0077 | | 0.8351 | | 2.99E-08 | | 31.0397 | 0.0006 | 56284 |
| rs11934731 | 4 | 88831249 | | A/G | | -0.0674 | 0.0061 | | 0.6738 | | 8.39E-29 | | 122.08 | 0.0022 | 56284 |
| rs12044944 | 1 | 240581653 | | T/C | | 0.0553 | 0.0074 | | 0.1916 | | 7.54E-14 | | 55.8433 | 0.001 | 56284 |
| rs12258451 | 10 | 54423853 | | G/C | | -0.0702 | 0.0089 | | 0.1307 | | 2.41E-15 | | 62.2127 | 0.0011 | 56284 |
| rs12442242 | 15 | 38340874 | | G/A | | 0.0509 | 0.0082 | | 0.1499 | | 4.94E-10 | | 38.5294 | 0.0007 | 56284 |
| rs12534510 | 7 | 120730944 | | C/A | | 0.0395 | 0.0057 | | 0.5545 | | 3.15E-12 | | 48.0208 | 0.0009 | 56284 |
| rs12612325 | 2 | 119632252 | | A/G | | -0.0548 | 0.0078 | | 0.2122 | | 1.98E-12 | | 49.3579 | 0.0009 | 56284 |
| rs1286150 | 14 | 91464890 | | C/T | | 0.0549 | 0.0072 | | 0.1955 | | 2.44E-14 | | 58.1386 | 0.001 | 56284 |
| rs13204965 | 6 | 127167072 | | C/A | | -0.0619 | 0.007 | | 0.229 | | 1.02E-18 | | 78.1933 | 0.0014 | 56284 |
| rs143187557 | 11 | 47284279 | | T/C | | -0.1237 | 0.0203 | | 0.0223 | | 1.15E-09 | | 37.1306 | 0.0007 | 56284 |
| rs144279715 | 2 | 119548256 | | G/A | | 0.2295 | 0.0294 | | 0.0147 | | 6.18E-15 | | 60.9334 | 0.0011 | 56284 |
| rs144691710 | 17 | 41819562 | | G/A | | 0.1017 | 0.0113 | | 0.0751 | | 2.24E-19 | | 80.9971 | 0.0014 | 56284 |
| rs1452102 | 21 | 28773868 | | G/T | | 0.0345 | 0.0057 | | 0.4129 | | 1.74E-09 | | 36.633 | 0.0007 | 56284 |
| rs1548607 | 7 | 50901491 | | G/A | | -0.0363 | 0.0066 | | 0.313 | | 4.18E-08 | | 30.2489 | 0.0005 | 56284 |
| rs2252865 | 1 | 8422676 | | C/T | | 0.0328 | 0.006 | | 0.6759 | | 4.72E-08 | | 29.8834 | 0.0005 | 56284 |
| rs2289410 | 2 | 42284110 | | T/A | | -0.0494 | 0.0088 | | 0.132 | | 2.00E-08 | | 31.5118 | 0.0006 | 56284 |
| rs2350085 | 2 | 202799604 | | C/T | | 0.0643 | 0.0085 | | 0.1274 | | 3.79E-14 | | 57.2227 | 0.001 | 56284 |
| rs2414098 | 15 | 51537806 | | C/T | | 0.0329 | 0.0059 | | 0.6102 | | 1.99E-08 | | 31.0937 | 0.0006 | 56284 |
| rs2566751 | 1 | 68664913 | | A/T | | -0.0567 | 0.01 | | 0.8727 | | 1.32E-08 | | 32.1478 | 0.0006 | 56284 |
| rs2566752 | 1 | 68656697 | | C/T | | 0.0721 | 0.0059 | | 0.3897 | | 1.88E-34 | | 149.3314 | 0.0026 | 56284 |
| rs2873195 | 17 | 2064702 | | T/A | | 0.0406 | 0.0062 | | 0.6873 | | 4.31E-11 | | 42.8798 | 0.0008 | 56284 |
| rs34102936 | 7 | 38142840 | | A/G | | 0.0471 | 0.0057 | | 0.5897 | | 1.87E-16 | | 68.2774 | 0.0012 | 56284 |
| rs34670419 | 7 | 99130834 | | T/G | | -0.088 | 0.0154 | | 0.0394 | | 1.09E-08 | | 32.6519 | 0.0006 | 56284 |
| rs35125553 | 12 | 1639249 | | G/A | | 0.0383 | 0.0066 | | 0.2855 | | 5.20E-09 | | 33.674 | 0.0006 | 56284 |
| rs35199438 | 11 | 16630779 | | T/G | | -0.0489 | 0.0062 | | 0.3035 | | 2.36E-15 | | 62.2041 | 0.0011 | 56284 |
| rs3743347 | 15 | 67547301 | | A/C | | 0.0519 | 0.0068 | | 0.2351 | | 1.75E-14 | | 58.2507 | 0.001 | 56284 |
| rs3801387 | 7 | 120974765 | | G/A | | 0.1347 | 0.0063 | | 0.2721 | | 1.15E-100 | | 457.1289 | 0.0081 | 56284 |
| rs4757350 | 11 | 15703674 | | T/C | | -0.0564 | 0.0069 | | 0.7852 | | 3.75E-16 | | 66.8105 | 0.0012 | 56284 |
| rs4846580 | 1 | 219897941 | | A/G | | 0.0345 | 0.0058 | | 0.5329 | | 3.21E-09 | | 35.3807 | 0.0006 | 56284 |
| rs55781332 | 11 | 242859 | | G/A | | 0.0552 | 0.0069 | | 0.2169 | | 8.07E-16 | | 63.9977 | 0.0011 | 56284 |
| rs56104760 | 1 | 22486029 | | G/A | | -0.0747 | 0.0074 | | 0.1905 | | 7.38E-24 | | 101.8972 | 0.0018 | 56284 |
| rs6029130 | 20 | 39103882 | | T/C | | 0.0348 | 0.0063 | | 0.2874 | | 3.50E-08 | | 30.5114 | 0.0005 | 56284 |
| rs6040063 | 20 | 10640877 | | G/A | | -0.0359 | 0.0056 | | 0.4993 | | 1.78E-10 | | 41.0958 | 0.0007 | 56284 |
| rs61884327 | 11 | 46766890 | | C/T | | 0.0801 | 0.0099 | | 0.0978 | | 4.63E-16 | | 65.4605 | 0.0012 | 56284 |
| rs633995 | 1 | 172186729 | | A/G | | 0.0351 | 0.0058 | | 0.4251 | | 1.61E-09 | | 36.6221 | 0.0007 | 56284 |
| rs634277 | 11 | 86887931 | | G/A | | -0.0607 | 0.0061 | | 0.3322 | | 2.15E-23 | | 99.0153 | 0.0018 | 56284 |
| rs6465511 | 7 | 96134115 | | G/C | | 0.0738 | 0.006 | | 0.6752 | | 1.03E-34 | | 151.2846 | 0.0027 | 56284 |
| rs6557155 | 6 | 151910126 | | G/T | | 0.0751 | 0.0059 | | 0.5682 | | 2.56E-37 | | 162.0169 | 0.0029 | 56284 |
| rs6960249 | 7 | 96660132 | | G/T | | -0.0325 | 0.0057 | | 0.4091 | | 1.45E-08 | | 32.5088 | 0.0006 | 56284 |
| rs71390846 | 16 | 86714715 | | C/G | | -0.0484 | 0.0075 | | 0.1836 | | 1.38E-10 | | 41.644 | 0.0007 | 56284 |
| rs725670 | 11 | 121913230 | | A/G | | -0.0322 | 0.0059 | | 0.383 | | 3.61E-08 | | 29.7846 | 0.0005 | 56284 |
| rs73169678 | 7 | 150953205 | | A/C | | 0.0619 | 0.0091 | | 0.1117 | | 1.05E-11 | | 46.2683 | 0.0008 | 56284 |
| rs73305797 | 7 | 30997087 | | T/A | | -0.0422 | 0.0067 | | 0.2431 | | 2.40E-10 | | 39.6698 | 0.0007 | 56284 |
| rs73349318 | 10 | 112245400 | | T/A | | 0.0472 | 0.0085 | | 0.1262 | | 2.68E-08 | | 30.8341 | 0.0005 | 56284 |
| rs73719807 | 7 | 121191251 | | C/A | | 0.0925 | 0.0112 | | 0.0871 | | 1.14E-16 | | 68.2075 | 0.0012 | 56284 |
| rs74394007 | 3 | 156692207 | | C/A | | -0.0608 | 0.0083 | | 0.138 | | 2.46E-13 | | 53.6581 | 0.001 | 56284 |
| rs7548588 | 1 | 110475971 | | C/T | | 0.0367 | 0.0058 | | 0.391 | | 2.21E-10 | | 40.0369 | 0.0007 | 56284 |
| rs757138 | 7 | 27989403 | | G/T | | 0.0348 | 0.0063 | | 0.3111 | | 3.33E-08 | | 30.5114 | 0.0005 | 56284 |
| rs7586085 | 2 | 166577489 | | G/A | | -0.0532 | 0.0057 | | 0.4663 | | 8.64E-21 | | 87.108 | 0.0015 | 56284 |
| rs76051363 | 4 | 1006987 | | T/C | | -0.0794 | 0.0085 | | 0.1491 | | 1.39E-20 | | 87.2545 | 0.0015 | 56284 |
| rs7728694 | 5 | 88288341 | | T/G | | -0.0503 | 0.0059 | | 0.461 | | 1.30E-17 | | 72.6803 | 0.0013 | 56284 |
| rs7740042 | 6 | 151971720 | | A/T | | -0.0494 | 0.0071 | | 0.2024 | | 2.71E-12 | | 48.4085 | 0.0009 | 56284 |
| rs7741085 | 6 | 44636919 | | T/C | | 0.0423 | 0.0057 | | 0.5874 | | 1.51E-13 | | 55.0701 | 0.001 | 56284 |
| rs78667121 | 13 | 43200103 | | A/G | | 0.1326 | 0.018 | | 0.0325 | | 1.70E-13 | | 54.2658 | 0.001 | 56284 |
| rs8047501 | 16 | 392318 | | G/A | | -0.0524 | 0.0059 | | 0.5077 | | 1.13E-18 | | 78.8757 | 0.0014 | 56284 |
| rs8070128 | 17 | 17804725 | | T/C | | -0.0394 | 0.0059 | | 0.5763 | | 1.98E-11 | | 44.5936 | 0.0008 | 56284 |
| rs818427 | 5 | 112221869 | | T/C | | 0.0342 | 0.0061 | | 0.3118 | | 2.37E-08 | | 31.4324 | 0.0006 | 56284 |
| rs838721 | 2 | 234308782 | | G/A | | 0.0314 | 0.0057 | | 0.563 | | 4.48E-08 | | 30.3455 | 0.0005 | 56284 |
| rs884205 | 18 | 60054857 | | C/A | | 0.0531 | 0.0068 | | 0.7579 | | 4.39E-15 | | 60.9756 | 0.0011 | 56284 |
| rs9594738 | 13 | 42952145 | | T/C | | -0.0614 | 0.0057 | | 0.4592 | | 3.84E-27 | | 116.0303 | 0.0021 | 56284 |
| rs9910055 | 17 | 42283037 | | T/C | | 0.0442 | 0.0067 | | 0.2624 | | 3.12E-11 | | 43.5191 | 0.0008 | 56284 |
| rs9972944 | 17 | 63771079 | | G/A | | -0.0363 | 0.0059 | | 0.5951 | | 6.87E-10 | | 37.8524 | 0.0007 | 56284 |
| rs9976876 | 21 | 36970350 | | T/G | | -0.0375 | 0.0058 | | 0.447 | | 8.01E-11 | | 41.8014 | 0.0007 | 56284 |
| **Genetic variants of Femoral neck bone mineral density** | | | | | | | |  |  |  |  |  |  |  |  |
| rs10170839 | 2 | 166572906 | | C/A | | -0.059358 | 0.007521 | | 0.431424 | | 1.20E-14 | | 62.2859 | 0.0012 | 49988 |
| rs10794639 | 16 | 377367 | | G/A | | -0.051188 | 0.007546 | | 0.458313 | | 3.30E-11 | | 46.0135 | 0.0009 | 49988 |
| rs10946458 | 6 | 21391282 | | C/T | | -0.044918 | 0.007974 | | 0.338425 | | 3.63E-08 | | 31.7301 | 0.0006 | 49988 |
| rs11652763 | 17 | 42208172 | | A/G | | 0.083568 | 0.012659 | | 0.147574 | | 1.09E-10 | | 43.5777 | 0.0009 | 49988 |
| rs13194508 | 6 | 127157438 | | C/T | | -0.051843 | 0.008915 | | 0.195465 | | 1.30E-08 | | 33.8158 | 0.0007 | 49988 |
| rs1366594 | 5 | 88376061 | | C/A | | -0.079453 | 0.007525 | | 0.522593 | | 5.44E-25 | | 111.4783 | 0.0022 | 49988 |
| rs1485307 | 8 | 120007395 | | C/T | | -0.061597 | 0.00761 | | 0.582657 | | 2.49E-15 | | 65.5137 | 0.0013 | 49988 |
| rs1785493 | 11 | 68208345 | | T/C | | -0.045109 | 0.008037 | | 0.365235 | | 4.06E-08 | | 31.5008 | 0.0006 | 49988 |
| rs2566752 | 1 | 68656697 | | C/T | | 0.061943 | 0.007699 | | 0.41074 | | 3.65E-15 | | 64.7289 | 0.0013 | 49988 |
| rs2741856 | 17 | 41826839 | | C/G | | 0.087631 | 0.014131 | | 0.0573588 | | 1.34E-09 | | 38.455 | 0.0008 | 49988 |
| rs3779381 | 7 | 120966790 | | G/A | | 0.057989 | 0.008522 | | 0.252188 | | 2.87E-11 | | 46.301 | 0.0009 | 49988 |
| rs4281029 | 7 | 38153807 | | A/C | | 0.056816 | 0.009361 | | 0.187749 | | 2.96E-09 | | 36.8366 | 0.0007 | 49988 |
| rs436448 | 3 | 41121251 | | C/T | | 0.063845 | 0.007565 | | 0.519809 | | 1.56E-16 | | 71.2227 | 0.0014 | 49988 |
| rs4448201 | 7 | 96154912 | | C/G | | 0.065631 | 0.007895 | | 0.700398 | | 4.37E-16 | | 69.1029 | 0.0014 | 49988 |
| rs4759320 | 12 | 54433011 | | C/G | | -0.044812 | 0.007933 | | 0.334049 | | 3.33E-08 | | 31.9078 | 0.0006 | 49988 |
| rs7108738 | 11 | 15710084 | | G/T | | 0.082771 | 0.009715 | | 0.192681 | | 8.07E-17 | | 72.5861 | 0.0015 | 49988 |
| rs71390846 | 16 | 86714715 | | C/G | | -0.059106 | 0.009756 | | 0.173667 | | 3.16E-09 | | 36.7031 | 0.0007 | 49988 |
| rs7209460 | 17 | 2048713 | | T/C | | 0.050829 | 0.008199 | | 0.705251 | | 1.35E-09 | | 38.4312 | 0.0008 | 49988 |
| rs7524102 | 1 | 22698447 | | G/A | | 0.083798 | 0.009823 | | 0.198329 | | 7.36E-17 | | 72.7716 | 0.0015 | 49988 |
| rs7524102 | 6 | 151874122 | | G/A | | 0.052736 | 0.007608 | | 0.482657 | | 1.23E-11 | | 48.0459 | 0.001 | 49988 |
| **Genetic variants of Forearm bone mineral density** | | | | | | | | | | | | | | | |
| rs13423976 | 2 | 38745624 | | G/A | | 0.097978 | 0.017179 | | 0.714002 | | 2.30E-08 | | 32.5223 | 0.003 | 10805 |
| rs6894139 | 5 | 88327782 | | G/T | | -0.088937 | 0.015638 | | 0.513206 | | 2.52E-08 | | 32.3387 | 0.003 | 10805 |
| rs7776725 | 7 | 121033121 | | C/T | | 0.186109 | 0.017421 | | 0.259348 | | 1.21E-25 | | 114.1059 | 0.0105 | 10805 |
| **Genetic variants of Heel bone mineral density** | | | | | | | | | |  |  |  |  |  |  |
| rs10069783 | 5 | 111985214 | | G/A | | 0.0169756 | 0.0030398 | | 0.231945 | | 2.30E-08 | | 31.1858 | 0.0001 | 265627 |
| rs10078310 | 5 | 54832387 | | A/G | | 0.0319733 | 0.00470271 | | 0.080426 | | 1.10E-11 | | 46.2248 | 0.0002 | 265627 |
| rs10221698 | 2 | 202939654 | | C/T | | 0.018198 | 0.00256676 | | 0.558609 | | 1.30E-12 | | 50.2659 | 0.0002 | 265627 |
| rs10235021 | 7 | 38123667 | | T/C | | 0.0715592 | 0.0031732 | | 0.201397 | | 1.30E-112 | | 508.549 | 0.0019 | 265627 |
| rs10239787 | 7 | 27970153 | | T/C | | -0.0283749 | 0.00271854 | | 0.329165 | | 1.70E-25 | | 108.9417 | 0.0004 | 265627 |
| rs10249754 | 7 | 15693906 | | G/A | | 0.0305864 | 0.00268284 | | 0.655244 | | 4.10E-30 | | 129.9762 | 0.0005 | 265627 |
| rs1029830 | 17 | 17184097 | | A/C | | -0.0166726 | 0.00255765 | | 0.500114 | | 7.10E-11 | | 42.4934 | 0.0002 | 265627 |
| rs1042704 | 14 | 23312594 | | A/G | | -0.0303501 | 0.00312359 | | 0.214121 | | 2.60E-22 | | 94.408 | 0.0004 | 265627 |
| rs10455097 | 6 | 74493432 | | C/A | | -0.0257736 | 0.00254875 | | 0.514317 | | 4.90E-24 | | 102.2569 | 0.0004 | 265627 |
| rs10473282 | 5 | 42410060 | | A/G | | -0.0194845 | 0.00265936 | | 0.365853 | | 2.40E-13 | | 53.681 | 0.0002 | 265627 |
| rs10491280 | 5 | 132196383 | | C/A | | 0.0222228 | 0.00365521 | | 0.14278 | | 1.20E-09 | | 36.9632 | 0.0001 | 265627 |
| rs10740042 | 10 | 62496586 | | C/T | | 0.0159156 | 0.00277138 | | 0.691373 | | 9.30E-09 | | 32.98 | 0.0001 | 265627 |
| rs10750766 | 11 | 65473798 | | A/C | | 0.024465 | 0.00282164 | | 0.710185 | | 4.30E-18 | | 75.1768 | 0.0003 | 265627 |
| rs10765568 | 11 | 92607866 | | T/C | | -0.017467 | 0.00264442 | | 0.373206 | | 4.00E-11 | | 43.6287 | 0.0002 | 265627 |
| rs10777536 | 12 | 94120732 | | A/G | | 0.0164167 | 0.002566 | | 0.511584 | | 1.60E-10 | | 40.9313 | 0.0002 | 265627 |
| rs10821027 | 9 | 95607083 | | T/C | | -0.017406 | 0.00259517 | | 0.417488 | | 2.00E-11 | | 44.9845 | 0.0002 | 265627 |
| rs10842704 | 12 | 26462982 | | G/T | | 0.0248457 | 0.00300942 | | 0.235836 | | 1.50E-16 | | 68.1606 | 0.0003 | 265627 |
| rs10922489 | 1 | 89259862 | | T/C | | -0.0151833 | 0.00255522 | | 0.542301 | | 2.80E-09 | | 35.3079 | 0.0001 | 265627 |
| rs10931982 | 2 | 202832130 | | C/T | | 0.0517236 | 0.00305416 | | 0.77443 | | 2.50E-64 | | 286.8076 | 0.0011 | 265627 |
| rs10932006 | 2 | 204001724 | | A/G | | -0.0532819 | 0.00751671 | | 0.970544 | | 1.40E-12 | | 50.2459 | 0.0002 | 265627 |
| rs10992875 | 9 | 96481284 | | T/C | | 0.0287334 | 0.00292078 | | 0.261425 | | 7.80E-23 | | 96.7772 | 0.0004 | 265627 |
| rs11067228 | 12 | 115094260 | | G/A | | 0.016852 | 0.0025643 | | 0.450275 | | 5.00E-11 | | 43.1879 | 0.0002 | 265627 |
| rs11088458 | 21 | 40350120 | | G/A | | -0.0418906 | 0.00285645 | | 0.713363 | | 1.10E-48 | | 215.0684 | 0.0008 | 265627 |
| rs11175773 | 12 | 65861817 | | T/C | | 0.0250208 | 0.00444426 | | 0.094707 | | 1.80E-08 | | 31.6957 | 0.0001 | 265627 |
| rs11175835 | 12 | 65995687 | | A/G | | -0.0242807 | 0.00282896 | | 0.71306 | | 9.30E-18 | | 73.6657 | 0.0003 | 265627 |
| rs111838776 | 11 | 48889461 | | T/A | | -0.0488755 | 0.00868979 | | 0.024369 | | 1.90E-08 | | 31.6345 | 0.0001 | 265627 |
| rs11214530 | 11 | 113045684 | | G/A | | -0.096705 | 0.00948641 | | 0.018458 | | 2.10E-24 | | 103.918 | 0.0004 | 265627 |
| rs11228240 | 11 | 68218290 | | T/C | | -0.0405934 | 0.00285797 | | 0.276559 | | 8.70E-46 | | 201.7401 | 0.0008 | 265627 |
| rs11229574 | 11 | 58435310 | | T/C | | 0.0173712 | 0.00299903 | | 0.240544 | | 6.90E-09 | | 33.5502 | 0.0001 | 265627 |
| rs1133400 | 10 | 134459388 | | G/A | | 0.0290652 | 0.0030855 | | 0.219311 | | 4.50E-21 | | 88.7344 | 0.0003 | 265627 |
| rs113869252 | 2 | 145797829 | | T/C | | 0.0170242 | 0.0029174 | | 0.255804 | | 5.40E-09 | | 34.0517 | 0.0001 | 265627 |
| rs114658808 | 2 | 119423996 | | A/G | | 0.0581104 | 0.00833813 | | 0.024877 | | 3.20E-12 | | 48.5699 | 0.0002 | 265627 |
| rs11576308 | 1 | 103385373 | | A/G | | 0.0166844 | 0.00261755 | | 0.607996 | | 1.80E-10 | | 40.6282 | 0.0002 | 265627 |
| rs1159798 | 10 | 54412493 | | C/A | | -0.0643859 | 0.00305331 | | 0.77501 | | 1.00E-98 | | 444.6686 | 0.0017 | 265627 |
| rs116208713 | 4 | 86917834 | | G/T | | 0.0293031 | 0.00490864 | | 0.072865 | | 2.40E-09 | | 35.637 | 0.0001 | 265627 |
| rs116228246 | 6 | 7050935 | | C/G | | 0.0879306 | 0.00853445 | | 0.023284 | | 6.80E-25 | | 106.1514 | 0.0004 | 265627 |
| rs11657636 | 17 | 2053920 | | T/C | | 0.0511577 | 0.00278587 | | 0.697743 | | 2.60E-75 | | 337.2074 | 0.0013 | 265627 |
| rs11688424 | 2 | 159884324 | | T/C | | 0.0204691 | 0.00262953 | | 0.374202 | | 7.00E-15 | | 60.5952 | 0.0002 | 265627 |
| rs11696009 | 2 | 72330334 | | C/A | | 0.0168531 | 0.00269415 | | 0.343814 | | 4.00E-10 | | 39.1303 | 0.0001 | 265627 |
| rs117111740 | 11 | 62201239 | | C/T | | -0.105151 | 0.00804878 | | 0.026784 | | 5.30E-39 | | 170.6725 | 0.0006 | 265627 |
| rs117124857 | 7 | 121009802 | | T/C | | -0.09406 | 0.00905747 | | 0.02226 | | 2.90E-25 | | 107.8432 | 0.0004 | 265627 |
| rs11729023 | 4 | 71991184 | | T/C | | 0.0263577 | 0.00393426 | | 0.12091 | | 2.10E-11 | | 44.8834 | 0.0002 | 265627 |
| rs117445182 | 6 | 151730166 | | A/C | | -0.0703227 | 0.00988322 | | 0.017777 | | 1.10E-12 | | 50.628 | 0.0002 | 265627 |
| rs117481343 | 12 | 13328208 | | T/C | | 0.133565 | 0.00786615 | | 0.02882 | | 1.20E-64 | | 288.3086 | 0.0011 | 265627 |
| rs118115924 | 12 | 49379537 | | T/G | | -0.191358 | 0.0119281 | | 0.011552 | | 6.40E-58 | | 257.3638 | 0.001 | 265627 |
| rs11904619 | 2 | 233720166 | | C/T | | -0.0178864 | 0.00317791 | | 0.20051 | | 1.80E-08 | | 31.6781 | 0.0001 | 265627 |
| rs11915970 | 3 | 56169965 | | T/A | | 0.0426355 | 0.00396919 | | 0.118258 | | 6.50E-27 | | 115.3814 | 0.0004 | 265627 |
| rs11947360 | 4 | 77874144 | | A/G | | -0.0205614 | 0.00354677 | | 0.153939 | | 6.70E-09 | | 33.6075 | 0.0001 | 265627 |
| rs11985969 | 8 | 25311777 | | A/G | | 0.0171364 | 0.00278274 | | 0.302841 | | 7.40E-10 | | 37.9219 | 0.0001 | 265627 |
| rs11993876 | 8 | 72218617 | | C/T | | 0.0150285 | 0.00259125 | | 0.42918 | | 6.60E-09 | | 33.6364 | 0.0001 | 265627 |
| rs12127020 | 1 | 68404391 | | G/C | | 0.0193384 | 0.00325031 | | 0.190536 | | 2.70E-09 | | 35.3988 | 0.0001 | 265627 |
| rs12145525 | 1 | 6711146 | | C/T | | -0.0205456 | 0.00268353 | | 0.346316 | | 1.90E-14 | | 58.6167 | 0.0002 | 265627 |
| rs12276167 | 11 | 27635571 | | G/T | | -0.0167977 | 0.0026068 | | 0.592974 | | 1.20E-10 | | 41.5223 | 0.0002 | 265627 |
| rs12340775 | 9 | 13226945 | | A/G | | -0.037198 | 0.00574106 | | 0.055719 | | 9.20E-11 | | 41.9809 | 0.0002 | 265627 |
| rs12403844 | 1 | 172214642 | | G/A | | 0.0278896 | 0.00309927 | | 0.214575 | | 2.30E-19 | | 80.9771 | 0.0003 | 265627 |
| rs12443252 | 15 | 91076028 | | T/C | | 0.0232857 | 0.00258405 | | 0.549556 | | 2.00E-19 | | 81.2033 | 0.0003 | 265627 |
| rs12448684 | 16 | 81580088 | | A/G | | -0.0251996 | 0.00256611 | | 0.488286 | | 9.20E-23 | | 96.4347 | 0.0004 | 265627 |
| rs12614608 | 2 | 153331546 | | A/G | | -0.0199646 | 0.00285024 | | 0.276731 | | 2.50E-12 | | 49.0631 | 0.0002 | 265627 |
| rs12622690 | 2 | 119086841 | | T/C | | -0.0468064 | 0.00285109 | | 0.723031 | | 1.40E-60 | | 269.5165 | 0.001 | 265627 |
| rs12636449 | 3 | 50180613 | | G/A | | 0.0435997 | 0.00457611 | | 0.085448 | | 1.60E-21 | | 90.776 | 0.0003 | 265627 |
| rs12714240 | 2 | 28941150 | | A/G | | -0.0209999 | 0.00265908 | | 0.641166 | | 2.80E-15 | | 62.3689 | 0.0002 | 265627 |
| rs12733821 | 1 | 162086691 | | C/G | | 0.0181773 | 0.00266114 | | 0.646405 | | 8.50E-12 | | 46.6573 | 0.0002 | 265627 |
| rs12761013 | 10 | 123635066 | | T/C | | 0.0159492 | 0.00268087 | | 0.348113 | | 2.70E-09 | | 35.3935 | 0.0001 | 265627 |
| rs1286077 | 14 | 91446384 | | C/T | | 0.0324697 | 0.00339615 | | 0.172515 | | 1.20E-21 | | 91.407 | 0.0003 | 265627 |
| rs12915039 | 15 | 67434348 | | C/A | | 0.019891 | 0.00303345 | | 0.240277 | | 5.50E-11 | | 42.9968 | 0.0002 | 265627 |
| rs12951408 | 17 | 36913807 | | C/T | | -0.0145193 | 0.00258215 | | 0.559368 | | 1.90E-08 | | 31.6173 | 0.0001 | 265627 |
| rs12975920 | 19 | 41793137 | | T/C | | 0.021043 | 0.00301396 | | 0.237351 | | 2.90E-12 | | 48.7458 | 0.0002 | 265627 |
| rs12985258 | 19 | 2159571 | | C/T | | -0.0190114 | 0.00262453 | | 0.400248 | | 4.40E-13 | | 52.4713 | 0.0002 | 265627 |
| rs13022041 | 2 | 25400559 | | T/G | | 0.0177883 | 0.00272968 | | 0.325615 | | 7.20E-11 | | 42.4661 | 0.0002 | 265627 |
| rs13044413 | 20 | 33020957 | | G/A | | 0.0205148 | 0.00258521 | | 0.440373 | | 2.10E-15 | | 62.9709 | 0.0002 | 265627 |
| rs13088318 | 3 | 101242751 | | G/A | | 0.015686 | 0.00270186 | | 0.33694 | | 6.40E-09 | | 33.7051 | 0.0001 | 265627 |
| rs13133616 | 4 | 157505233 | | G/A | | 0.0175101 | 0.00293914 | | 0.254309 | | 2.60E-09 | | 35.4922 | 0.0001 | 265627 |
| rs13220896 | 6 | 31369662 | | A/G | | -0.0452947 | 0.00642699 | | 0.040608 | | 1.80E-12 | | 49.668 | 0.0002 | 265627 |
| rs132530 | 22 | 29296931 | | A/C | | 0.0507039 | 0.00723676 | | 0.967624 | | 2.40E-12 | | 49.0898 | 0.0002 | 265627 |
| rs13264707 | 8 | 129950004 | | C/T | | 0.0151932 | 0.00263902 | | 0.38356 | | 8.60E-09 | | 33.1444 | 0.0001 | 265627 |
| rs13379337 | 14 | 35224430 | | A/C | | 0.0199008 | 0.0025731 | | 0.488789 | | 1.00E-14 | | 59.817 | 0.0002 | 265627 |
| rs1381957 | 4 | 88796699 | | G/A | | -0.0434382 | 0.00363757 | | 0.855846 | | 7.20E-33 | | 142.5994 | 0.0005 | 265627 |
| rs1386625 | 4 | 38361120 | | G/A | | -0.0475961 | 0.00436858 | | 0.903038 | | 1.20E-27 | | 118.7022 | 0.0004 | 265627 |
| rs139603701 | 1 | 2904634 | | G/A | | -0.105562 | 0.0103602 | | 0.016446 | | 2.20E-24 | | 103.8187 | 0.0004 | 265627 |
| rs1414660 | 1 | 240586695 | | T/C | | 0.0813511 | 0.00322854 | | 0.19205 | | 4.30E-140 | | 634.9087 | 0.0024 | 265627 |
| rs141795717 | 2 | 119539208 | | A/G | | 0.224255 | 0.0100799 | | 0.016396 | | 1.20E-109 | | 494.9582 | 0.0019 | 265627 |
| rs1420996 | 16 | 51422686 | | T/C | | 0.0162034 | 0.00292174 | | 0.738004 | | 2.90E-08 | | 30.7557 | 0.0001 | 265627 |
| rs143348825 | 12 | 49248340 | | A/G | | -0.0577003 | 0.00995869 | | 0.017037 | | 6.90E-09 | | 33.5698 | 0.0001 | 265627 |
| rs1444436 | 1 | 163921487 | | G/C | | -0.016483 | 0.00276025 | | 0.690709 | | 2.40E-09 | | 35.6593 | 0.0001 | 265627 |
| rs1461930 | 16 | 51719647 | | T/C | | 0.0315967 | 0.00305487 | | 0.227358 | | 4.50E-25 | | 106.9781 | 0.0004 | 265627 |
| rs1502201 | 6 | 55265497 | | A/G | | -0.0181193 | 0.00292692 | | 0.747177 | | 6.00E-10 | | 38.3229 | 0.0001 | 265627 |
| rs150445982 | 6 | 55676621 | | T/C | | 0.0839094 | 0.00892844 | | 0.022806 | | 5.60E-21 | | 88.3216 | 0.0003 | 265627 |
| rs150967545 | 7 | 121121903 | | A/G | | 0.0450273 | 0.00756982 | | 0.032425 | | 2.70E-09 | | 35.3816 | 0.0001 | 265627 |
| rs1533142 | 12 | 28614139 | | G/C | | -0.0255772 | 0.00304406 | | 0.771962 | | 4.40E-17 | | 70.5986 | 0.0003 | 265627 |
| rs1535570 | 10 | 77621732 | | A/C | | 0.0168272 | 0.00257632 | | 0.562649 | | 6.50E-11 | | 42.66 | 0.0002 | 265627 |
| rs1555958 | 20 | 10976046 | | G/C | | 0.0271 | 0.00276317 | | 0.68358 | | 1.00E-22 | | 96.1878 | 0.0004 | 265627 |
| rs1568609 | 3 | 157066839 | | A/G | | -0.014586 | 0.00264047 | | 0.621199 | | 3.30E-08 | | 30.5145 | 0.0001 | 265627 |
| rs159408 | 6 | 133702841 | | T/A | | -0.0147223 | 0.00259889 | | 0.407017 | | 1.50E-08 | | 32.0902 | 0.0001 | 265627 |
| rs1622638 | 11 | 121800971 | | A/G | | 0.0179885 | 0.00261201 | | 0.394463 | | 5.70E-12 | | 47.4282 | 0.0002 | 265627 |
| rs1644335 | 19 | 47272422 | | A/G | | -0.020498 | 0.00364692 | | 0.144929 | | 1.90E-08 | | 31.5913 | 0.0001 | 265627 |
| rs1649082 | 10 | 60292434 | | C/A | | -0.0171775 | 0.00256889 | | 0.541181 | | 2.30E-11 | | 44.7122 | 0.0002 | 265627 |
| rs16840232 | 1 | 240646061 | | C/T | | 0.0184235 | 0.00321822 | | 0.216243 | | 1.00E-08 | | 32.7725 | 0.0001 | 265627 |
| rs16857021 | 2 | 218070164 | | A/T | | 0.0283504 | 0.00349044 | | 0.159411 | | 4.60E-16 | | 65.9713 | 0.0002 | 265627 |
| rs16878921 | 5 | 50778798 | | A/G | | 0.0358608 | 0.00429292 | | 0.098068 | | 6.60E-17 | | 69.78 | 0.0003 | 265627 |
| rs16891104 | 4 | 14860842 | | C/T | | -0.0185824 | 0.00321193 | | 0.19653 | | 7.20E-09 | | 33.471 | 0.0001 | 265627 |
| rs1694593 | 1 | 220111743 | | G/A | | -0.028844 | 0.00315199 | | 0.794678 | | 5.60E-20 | | 83.741 | 0.0003 | 265627 |
| rs1706708 | 15 | 70552313 | | A/G | | -0.0273671 | 0.00273719 | | 0.327964 | | 1.60E-23 | | 99.9642 | 0.0004 | 265627 |
| rs17152567 | 7 | 25793477 | | G/C | | -0.0300244 | 0.00301611 | | 0.234582 | | 2.40E-23 | | 99.0948 | 0.0004 | 265627 |
| rs1716169 | 12 | 123716930 | | T/A | | 0.0206238 | 0.00319145 | | 0.798379 | | 1.00E-10 | | 41.7598 | 0.0002 | 265627 |
| rs17173698 | 10 | 89468953 | | A/G | | 0.0544865 | 0.00815467 | | 0.025086 | | 2.40E-11 | | 44.6439 | 0.0002 | 265627 |
| rs17457340 | 20 | 10640042 | | C/T | | -0.0677286 | 0.00482945 | | 0.07654 | | 1.10E-44 | | 196.6734 | 0.0007 | 265627 |
| rs17507577 | 11 | 15243059 | | A/G | | 0.0578242 | 0.00486329 | | 0.074154 | | 1.30E-32 | | 141.3695 | 0.0005 | 265627 |
| rs17601876 | 15 | 51553909 | | G/A | | 0.0303011 | 0.00256678 | | 0.478533 | | 3.70E-32 | | 139.3594 | 0.0005 | 265627 |
| rs17680862 | 16 | 67322118 | | C/G | | -0.0695834 | 0.00800535 | | 0.026167 | | 3.60E-18 | | 75.5522 | 0.0003 | 265627 |
| rs1784998 | 11 | 115536210 | | C/T | | -0.0371287 | 0.00399474 | | 0.883699 | | 1.50E-20 | | 86.3852 | 0.0003 | 265627 |
| rs1811291 | 5 | 154028817 | | G/A | | -0.0226969 | 0.00347224 | | 0.162742 | | 6.30E-11 | | 42.7278 | 0.0002 | 265627 |
| rs1834554 | 11 | 60007573 | | C/G | | -0.0224393 | 0.00337808 | | 0.173097 | | 3.10E-11 | | 44.1241 | 0.0002 | 265627 |
| rs1862947 | 7 | 50919937 | | C/T | | -0.0157338 | 0.00274393 | | 0.318643 | | 9.80E-09 | | 32.8789 | 0.0001 | 265627 |
| rs189267552 | 3 | 20073193 | | A/T | | -0.0756245 | 0.0115971 | | 0.01252 | | 7.00E-11 | | 42.5229 | 0.0002 | 265627 |
| rs190144 | 17 | 42164584 | | G/A | | -0.0346388 | 0.00293391 | | 0.744478 | | 3.60E-32 | | 139.3891 | 0.0005 | 265627 |
| rs1988961 | 17 | 59030129 | | C/T | | 0.0552146 | 0.00360814 | | 0.148226 | | 7.30E-53 | | 234.1735 | 0.0009 | 265627 |
| rs1991431 | 3 | 141133450 | | A/G | | -0.0152907 | 0.00257662 | | 0.441046 | | 2.90E-09 | | 35.2169 | 0.0001 | 265627 |
| rs2002810 | 7 | 95980004 | | C/T | | -0.023528 | 0.00276965 | | 0.68026 | | 2.00E-17 | | 72.1634 | 0.0003 | 265627 |
| rs2008411 | 13 | 99587929 | | T/C | | -0.0328864 | 0.00279105 | | 0.698707 | | 4.80E-32 | | 138.8335 | 0.0005 | 265627 |
| rs2052482 | 5 | 80282584 | | T/C | | 0.0256865 | 0.00283469 | | 0.285257 | | 1.30E-19 | | 82.1099 | 0.0003 | 265627 |
| rs2054810 | 6 | 72386408 | | G/A | | -0.0183198 | 0.00331712 | | 0.183766 | | 3.30E-08 | | 30.5011 | 0.0001 | 265627 |
| rs206432 | 18 | 10356076 | | T/G | | 0.0144512 | 0.00256786 | | 0.503818 | | 1.80E-08 | | 31.671 | 0.0001 | 265627 |
| rs2066829 | 9 | 98229389 | | G/C | | -0.0299274 | 0.00266885 | | 0.356554 | | 3.50E-29 | | 125.7437 | 0.0005 | 265627 |
| rs2069443 | 7 | 150755173 | | G/T | | -0.0177404 | 0.00291429 | | 0.255369 | | 1.10E-09 | | 37.056 | 0.0001 | 265627 |
| rs213759 | 1 | 41592998 | | A/G | | 0.0225796 | 0.00411467 | | 0.891056 | | 4.10E-08 | | 30.1134 | 0.0001 | 265627 |
| rs2146778 | 9 | 111133954 | | A/C | | 0.0197159 | 0.00303302 | | 0.235982 | | 8.00E-11 | | 42.2551 | 0.0002 | 265627 |
| rs215226 | 12 | 591300 | | G/A | | 0.0273506 | 0.00261731 | | 0.403353 | | 1.50E-25 | | 109.1994 | 0.0004 | 265627 |
| rs2174633 | 4 | 17917781 | | C/A | | 0.0200969 | 0.00290147 | | 0.735635 | | 4.30E-12 | | 47.9754 | 0.0002 | 265627 |
| rs2238688 | 19 | 46009520 | | C/G | | -0.0339243 | 0.00505212 | | 0.069158 | | 1.90E-11 | | 45.0891 | 0.0002 | 265627 |
| rs2242919 | 21 | 36576058 | | G/A | | 0.0190498 | 0.00337888 | | 0.176015 | | 1.70E-08 | | 31.7857 | 0.0001 | 265627 |
| rs2272443 | 16 | 88854008 | | T/C | | -0.0187135 | 0.00307289 | | 0.227955 | | 1.10E-09 | | 37.0862 | 0.0001 | 265627 |
| rs2296784 | 10 | 114723559 | | C/T | | -0.0263417 | 0.00314648 | | 0.214205 | | 5.70E-17 | | 70.0865 | 0.0003 | 265627 |
| rs239677 | 21 | 28774869 | | T/C | | -0.0215463 | 0.00261918 | | 0.409859 | | 1.90E-16 | | 67.6724 | 0.0003 | 265627 |
| rs2491105 | 9 | 130320283 | | C/T | | 0.0192308 | 0.00305573 | | 0.233606 | | 3.10E-10 | | 39.606 | 0.0001 | 265627 |
| rs2529015 | 7 | 20326586 | | T/C | | 0.0248267 | 0.00342616 | | 0.166064 | | 4.30E-13 | | 52.5073 | 0.0002 | 265627 |
| rs2531992 | 16 | 4021734 | | G/A | | 0.0255326 | 0.00354516 | | 0.845192 | | 5.90E-13 | | 51.8699 | 0.0002 | 265627 |
| rs2542710 | 5 | 39382261 | | G/A | | -0.030199 | 0.00256618 | | 0.484179 | | 5.70E-32 | | 138.4866 | 0.0005 | 265627 |
| rs2553772 | 11 | 35085453 | | G/T | | 0.0309456 | 0.002566 | | 0.540261 | | 1.70E-33 | | 145.4391 | 0.0005 | 265627 |
| rs2566752 | 1 | 68656697 | | C/T | | 0.0349158 | 0.00263874 | | 0.3759 | | 5.70E-40 | | 175.0844 | 0.0007 | 265627 |
| rs2566774 | 1 | 68694877 | | C/T | | -0.0383683 | 0.00326425 | | 0.811864 | | 6.70E-32 | | 138.1577 | 0.0005 | 265627 |
| rs2637252 | 10 | 78301598 | | G/A | | -0.0200251 | 0.00341637 | | 0.831669 | | 4.60E-09 | | 34.3571 | 0.0001 | 265627 |
| rs2663351 | 17 | 925006 | | C/T | | -0.0193795 | 0.00271418 | | 0.359069 | | 9.30E-13 | | 50.9806 | 0.0002 | 265627 |
| rs2716579 | 16 | 74087814 | | T/C | | -0.0265566 | 0.00311324 | | 0.356244 | | 1.50E-17 | | 72.764 | 0.0003 | 265627 |
| rs2722299 | 7 | 38013798 | | T/C | | -0.0250569 | 0.00258227 | | 0.427687 | | 2.90E-22 | | 94.156 | 0.0004 | 265627 |
| rs2737252 | 8 | 116663898 | | A/G | | 0.0356028 | 0.00284462 | | 0.281506 | | 6.10E-36 | | 156.645 | 0.0006 | 265627 |
| rs2812246 | 13 | 51309430 | | G/A | | 0.0264932 | 0.00432715 | | 0.09779 | | 9.20E-10 | | 37.4854 | 0.0001 | 265627 |
| rs28585071 | 6 | 168432467 | | A/G | | -0.0272259 | 0.00335692 | | 0.175655 | | 5.00E-16 | | 65.7778 | 0.0002 | 265627 |
| rs28706743 | 10 | 79394329 | | G/T | | -0.0296101 | 0.00334081 | | 0.178506 | | 7.80E-19 | | 78.5548 | 0.0003 | 265627 |
| rs2908007 | 7 | 120962164 | | G/A | | 0.164545 | 0.00259856 | | 0.401166 | | 1.00E-200 | | 4009.596 | 0.0149 | 265627 |
| rs2982573 | 6 | 152010534 | | C/T | | 0.0764832 | 0.0025796 | | 0.420225 | | 3.50E-193 | | 879.0713 | 0.0033 | 265627 |
| rs3099090 | 8 | 96666692 | | C/T | | -0.0225155 | 0.0030646 | | 0.772833 | | 2.00E-13 | | 53.9774 | 0.0002 | 265627 |
| rs3118906 | 13 | 51106788 | | A/G | | 0.0291031 | 0.00285664 | | 0.277737 | | 2.20E-24 | | 103.7921 | 0.0004 | 265627 |
| rs34137313 | 9 | 110893956 | | G/T | | -0.0233844 | 0.0030549 | | 0.227572 | | 1.90E-14 | | 58.5943 | 0.0002 | 265627 |
| rs34185333 | 7 | 27239193 | | T/C | | -0.0621689 | 0.0079194 | | 0.027889 | | 4.20E-15 | | 61.6252 | 0.0002 | 265627 |
| rs344035 | 3 | 156461279 | | A/G | | -0.0162519 | 0.00255953 | | 0.500099 | | 2.20E-10 | | 40.3167 | 0.0002 | 265627 |
| rs34441013 | 2 | 119632668 | | A/G | | 0.0339303 | 0.00285493 | | 0.277041 | | 1.40E-32 | | 141.2476 | 0.0005 | 265627 |
| rs347609 | 3 | 11269999 | | C/G | | -0.02532 | 0.00327586 | | 0.812157 | | 1.10E-14 | | 59.7411 | 0.0002 | 265627 |
| rs34965590 | 17 | 80015718 | | C/G | | -0.0146188 | 0.00266805 | | 0.634321 | | 4.30E-08 | | 30.0215 | 0.0001 | 265627 |
| rs35107139 | 14 | 54419106 | | C/A | | 0.0529374 | 0.00269798 | | 0.405016 | | 1.00E-85 | | 384.9856 | 0.0014 | 265627 |
| rs35172905 | 12 | 12424141 | | G/A | | 0.0212305 | 0.00335149 | | 0.177977 | | 2.40E-10 | | 40.1274 | 0.0002 | 265627 |
| rs35306535 | 11 | 68843990 | | A/G | | 0.0236213 | 0.0025527 | | 0.510192 | | 2.20E-20 | | 85.6258 | 0.0003 | 265627 |
| rs35531047 | 2 | 42220435 | | A/T | | 0.0514229 | 0.0030758 | | 0.221285 | | 9.60E-63 | | 279.5076 | 0.0011 | 265627 |
| rs368510 | 5 | 148787469 | | A/G | | 0.0271809 | 0.00270639 | | 0.333418 | | 9.80E-24 | | 100.8657 | 0.0004 | 265627 |
| rs370387 | 3 | 41123984 | | A/G | | 0.0424137 | 0.00256706 | | 0.554104 | | 2.50E-61 | | 272.9839 | 0.001 | 265627 |
| rs3730393 | 16 | 67226405 | | A/G | | 0.0368563 | 0.00654179 | | 0.040574 | | 1.80E-08 | | 31.7415 | 0.0001 | 265627 |
| rs3760456 | 17 | 27948844 | | T/C | | -0.023854 | 0.00257689 | | 0.439951 | | 2.10E-20 | | 85.6895 | 0.0003 | 265627 |
| rs3765971 | 1 | 8445360 | | T/C | | 0.0284684 | 0.00268359 | | 0.657495 | | 2.70E-26 | | 112.5358 | 0.0004 | 265627 |
| rs3775119 | 4 | 893712 | | T/C | | -0.0215573 | 0.00364475 | | 0.145037 | | 3.30E-09 | | 34.9824 | 0.0001 | 265627 |
| rs3777787 | 6 | 133577921 | | A/C | | -0.0504275 | 0.00255917 | | 0.541444 | | 2.00E-86 | | 388.2696 | 0.0015 | 265627 |
| rs3778929 | 7 | 156396471 | | C/T | | 0.0150937 | 0.00274437 | | 0.347275 | | 3.80E-08 | | 30.2484 | 0.0001 | 265627 |
| rs3790608 | 1 | 113055023 | | A/G | | 0.0327313 | 0.00356689 | | 0.151079 | | 4.50E-20 | | 84.2061 | 0.0003 | 265627 |
| rs3812849 | 13 | 74701736 | | C/A | | -0.0175127 | 0.00289987 | | 0.265604 | | 1.50E-09 | | 36.4709 | 0.0001 | 265627 |
| rs3843738 | 17 | 43739194 | | G/A | | 0.0167506 | 0.00259997 | | 0.470829 | | 1.20E-10 | | 41.5069 | 0.0002 | 265627 |
| rs3922468 | 5 | 95613007 | | A/G | | 0.0162468 | 0.00272496 | | 0.667951 | | 2.50E-09 | | 35.5478 | 0.0001 | 265627 |
| rs4053429 | 6 | 2488225 | | C/T | | -0.0245028 | 0.00255124 | | 0.521085 | | 7.70E-22 | | 92.2413 | 0.0003 | 265627 |
| rs41302867 | 6 | 7240876 | | A/G | | -0.0293283 | 0.00388156 | | 0.122612 | | 4.20E-14 | | 57.0897 | 0.0002 | 265627 |
| rs4148160 | 4 | 89015090 | | T/C | | 0.0181558 | 0.00265295 | | 0.374592 | | 7.70E-12 | | 46.8349 | 0.0002 | 265627 |
| rs42039 | 7 | 92244422 | | T/C | | -0.0248894 | 0.00297355 | | 0.243626 | | 5.70E-17 | | 70.0608 | 0.0003 | 265627 |
| rs42916 | 5 | 127560659 | | A/C | | 0.0229233 | 0.00291802 | | 0.739107 | | 4.00E-15 | | 61.7127 | 0.0002 | 265627 |
| rs4297774 | 18 | 77253456 | | G/T | | 0.0294179 | 0.00437843 | | 0.094978 | | 1.80E-11 | | 45.1423 | 0.0002 | 265627 |
| rs4304588 | 1 | 221476976 | | T/C | | -0.0235918 | 0.00260309 | | 0.40576 | | 1.30E-19 | | 82.1373 | 0.0003 | 265627 |
| rs4305309 | 2 | 54683711 | | C/T | | -0.0736623 | 0.0026656 | | 0.642791 | | 4.30E-168 | | 763.6552 | 0.0029 | 265627 |
| rs4505759 | 4 | 1003022 | | T/C | | 0.0555877 | 0.00278152 | | 0.307293 | | 7.50E-89 | | 399.3832 | 0.0015 | 265627 |
| rs4576968 | 13 | 42071635 | | G/A | | -0.0168212 | 0.00261996 | | 0.609827 | | 1.40E-10 | | 41.2213 | 0.0002 | 265627 |
| rs4595506 | 11 | 16542089 | | G/A | | -0.0234955 | 0.00255368 | | 0.494735 | | 3.60E-20 | | 84.6512 | 0.0003 | 265627 |
| rs4635400 | 18 | 13719510 | | A/G | | -0.0488982 | 0.00266865 | | 0.359201 | | 5.40E-75 | | 335.737 | 0.0013 | 265627 |
| rs4666343 | 2 | 19870341 | | T/C | | -0.0203756 | 0.00287039 | | 0.271619 | | 1.30E-12 | | 50.389 | 0.0002 | 265627 |
| rs4671961 | 2 | 54798596 | | A/G | | 0.0484689 | 0.0027342 | | 0.68074 | | 2.60E-70 | | 314.2407 | 0.0012 | 265627 |
| rs4782351 | 16 | 88466424 | | G/A | | -0.0164273 | 0.00262701 | | 0.611613 | | 4.00E-10 | | 39.1026 | 0.0001 | 265627 |
| rs4807630 | 19 | 1170445 | | T/C | | -0.0333307 | 0.00278927 | | 0.310033 | | 6.50E-33 | | 142.7922 | 0.0005 | 265627 |
| rs4820346 | 22 | 39125074 | | G/C | | 0.0211556 | 0.00278734 | | 0.693253 | | 3.20E-14 | | 57.606 | 0.0002 | 265627 |
| rs4870044 | 6 | 151901409 | | T/C | | -0.0802307 | 0.00280103 | | 0.289152 | | 1.90E-180 | | 820.4316 | 0.0031 | 265627 |
| rs4876858 | 8 | 119773438 | | C/G | | -0.0230877 | 0.00316328 | | 0.792869 | | 2.90E-13 | | 53.27 | 0.0002 | 265627 |
| rs4912084 | 1 | 19721548 | | G/A | | -0.0178066 | 0.00262965 | | 0.622386 | | 1.30E-11 | | 45.8525 | 0.0002 | 265627 |
| rs4940236 | 18 | 45604221 | | A/G | | 0.0167728 | 0.0026748 | | 0.355999 | | 3.60E-10 | | 39.3211 | 0.0001 | 265627 |
| rs55709850 | 22 | 50724733 | | T/G | | -0.0162812 | 0.0028384 | | 0.289644 | | 9.70E-09 | | 32.902 | 0.0001 | 265627 |
| rs55849728 | 12 | 90533915 | | T/C | | 0.0523158 | 0.00260341 | | 0.402124 | | 8.10E-90 | | 403.8102 | 0.0015 | 265627 |
| rs56257969 | 19 | 57797752 | | G/A | | -0.0160003 | 0.00292791 | | 0.259313 | | 4.60E-08 | | 29.8633 | 0.0001 | 265627 |
| rs56360935 | 17 | 43156135 | | T/A | | 0.0215866 | 0.00280655 | | 0.294475 | | 1.50E-14 | | 59.1588 | 0.0002 | 265627 |
| rs566046 | 11 | 86864277 | | G/A | | -0.0692625 | 0.00274629 | | 0.313586 | | 2.40E-140 | | 636.0632 | 0.0024 | 265627 |
| rs56921221 | 4 | 54352164 | | A/G | | 0.0257769 | 0.00359353 | | 0.149011 | | 7.30E-13 | | 51.4536 | 0.0002 | 265627 |
| rs571356 | 7 | 83306489 | | A/G | | -0.0182881 | 0.00273691 | | 0.318068 | | 2.40E-11 | | 44.6491 | 0.0002 | 265627 |
| rs57299708 | 2 | 42288320 | | T/C | | -0.0258833 | 0.0034543 | | 0.165738 | | 6.70E-14 | | 56.1456 | 0.0002 | 265627 |
| rs58552741 | 3 | 41016731 | | G/C | | -0.0270175 | 0.00436835 | | 0.105396 | | 6.20E-10 | | 38.2518 | 0.0001 | 265627 |
| rs59232853 | 14 | 53694054 | | C/T | | -0.0176905 | 0.00286928 | | 0.272919 | | 7.00E-10 | | 38.0129 | 0.0001 | 265627 |
| rs59429575 | 15 | 51607077 | | T/C | | 0.0207411 | 0.00294774 | | 0.261366 | | 2.00E-12 | | 49.5087 | 0.0002 | 265627 |
| rs59813731 | 19 | 18146068 | | T/C | | 0.0248479 | 0.00324347 | | 0.20116 | | 1.80E-14 | | 58.689 | 0.0002 | 265627 |
| rs60018147 | 19 | 3375572 | | G/A | | -0.0309803 | 0.0041361 | | 0.120313 | | 6.90E-14 | | 56.103 | 0.0002 | 265627 |
| rs603424 | 10 | 102075479 | | A/G | | -0.0264265 | 0.00336927 | | 0.172924 | | 4.40E-15 | | 61.5183 | 0.0002 | 265627 |
| rs6054184 | 20 | 6354592 | | G/A | | -0.0291131 | 0.0026323 | | 0.614158 | | 2.00E-28 | | 122.3215 | 0.0005 | 265627 |
| rs6059412 | 20 | 32280829 | | T/G | | 0.0204545 | 0.00282133 | | 0.29948 | | 4.20E-13 | | 52.5614 | 0.0002 | 265627 |
| rs6063051 | 20 | 45604420 | | G/T | | 0.029881 | 0.00293688 | | 0.741512 | | 2.60E-24 | | 103.5177 | 0.0004 | 265627 |
| rs60891864 | 17 | 54273598 | | G/C | | 0.0281682 | 0.00260892 | | 0.403094 | | 3.60E-27 | | 116.5718 | 0.0004 | 265627 |
| rs6117854 | 20 | 7551554 | | A/G | | -0.0376012 | 0.00275552 | | 0.325819 | | 2.10E-42 | | 186.2057 | 0.0007 | 265627 |
| rs6123756 | 20 | 56556146 | | C/A | | -0.0142865 | 0.00259692 | | 0.422108 | | 3.80E-08 | | 30.2643 | 0.0001 | 265627 |
| rs61843418 | 10 | 27912821 | | T/C | | -0.0190062 | 0.00312947 | | 0.214906 | | 1.30E-09 | | 36.8847 | 0.0001 | 265627 |
| rs61918361 | 11 | 102000753 | | T/C | | -0.0144539 | 0.00256547 | | 0.535989 | | 1.80E-08 | | 31.7419 | 0.0001 | 265627 |
| rs61921611 | 12 | 66367726 | | C/T | | 0.0192578 | 0.00277345 | | 0.30783 | | 3.80E-12 | | 48.2136 | 0.0002 | 265627 |
| rs62007686 | 14 | 103899344 | | G/A | | -0.0410633 | 0.00269543 | | 0.344827 | | 2.10E-52 | | 232.0857 | 0.0009 | 265627 |
| rs62028332 | 16 | 51025468 | | A/G | | 0.0437435 | 0.00382153 | | 0.146403 | | 2.40E-30 | | 131.0235 | 0.0005 | 265627 |
| rs62038775 | 16 | 11930010 | | G/A | | 0.0199821 | 0.0030409 | | 0.245893 | | 5.00E-11 | | 43.1792 | 0.0002 | 265627 |
| rs62178780 | 2 | 181929274 | | C/G | | 0.0218664 | 0.00278398 | | 0.296508 | | 4.00E-15 | | 61.6906 | 0.0002 | 265627 |
| rs62228064 | 22 | 46381414 | | A/G | | 0.0308867 | 0.00363112 | | 0.145917 | | 1.80E-17 | | 72.3533 | 0.0003 | 265627 |
| rs62331124 | 4 | 106034930 | | C/A | | 0.0176736 | 0.00276624 | | 0.313338 | | 1.70E-10 | | 40.8194 | 0.0002 | 265627 |
| rs635084 | 1 | 210466625 | | A/G | | 0.0275249 | 0.00335953 | | 0.174639 | | 2.50E-16 | | 67.1261 | 0.0003 | 265627 |
| rs6454314 | 6 | 83740400 | | A/G | | -0.0213809 | 0.00274257 | | 0.68457 | | 6.40E-15 | | 60.7762 | 0.0002 | 265627 |
| rs6475068 | 9 | 16693216 | | C/G | | 0.0316026 | 0.00429901 | | 0.901394 | | 2.00E-13 | | 54.0388 | 0.0002 | 265627 |
| rs6485702 | 11 | 46898771 | | C/T | | -0.0338887 | 0.00269443 | | 0.660327 | | 2.80E-36 | | 158.1877 | 0.0006 | 265627 |
| rs6532480 | 4 | 95277038 | | C/T | | -0.0155824 | 0.00258459 | | 0.43972 | | 1.70E-09 | | 36.3481 | 0.0001 | 265627 |
| rs660877 | 9 | 244577 | | C/G | | 0.0160236 | 0.00285223 | | 0.281149 | | 1.90E-08 | | 31.5608 | 0.0001 | 265627 |
| rs6650591 | 17 | 80508790 | | T/C | | 0.019289 | 0.00265601 | | 0.63304 | | 3.80E-13 | | 52.742 | 0.0002 | 265627 |
| rs6660927 | 1 | 21960044 | | C/A | | -0.0291881 | 0.00409502 | | 0.108804 | | 1.00E-12 | | 50.8038 | 0.0002 | 265627 |
| rs6684375 | 1 | 22706434 | | T/C | | 0.062122 | 0.00334042 | | 0.176265 | | 3.40E-77 | | 345.8481 | 0.0013 | 265627 |
| rs6716216 | 2 | 202803881 | | G/A | | 0.0353041 | 0.00389112 | | 0.122117 | | 1.20E-19 | | 82.3186 | 0.0003 | 265627 |
| rs672052 | 11 | 128661853 | | A/G | | 0.0151075 | 0.00271872 | | 0.333821 | | 2.70E-08 | | 30.8783 | 0.0001 | 265627 |
| rs6759927 | 2 | 238227919 | | G/A | | 0.0178931 | 0.00274013 | | 0.316093 | | 6.60E-11 | | 42.6408 | 0.0002 | 265627 |
| rs67631072 | 1 | 38461821 | | T/C | | 0.0180743 | 0.00257839 | | 0.553759 | | 2.40E-12 | | 49.1386 | 0.0002 | 265627 |
| rs67820526 | 19 | 4083916 | | A/G | | 0.0221764 | 0.0033794 | | 0.179407 | | 5.30E-11 | | 43.0625 | 0.0002 | 265627 |
| rs67918869 | 12 | 54394784 | | G/A | | 0.0211736 | 0.00340677 | | 0.170029 | | 5.10E-10 | | 38.6278 | 0.0001 | 265627 |
| rs68119427 | 4 | 57758030 | | G/C | | 0.0160556 | 0.00268933 | | 0.347618 | | 2.40E-09 | | 35.642 | 0.0001 | 265627 |
| rs6844904 | 4 | 146167657 | | A/G | | 0.0325072 | 0.00341855 | | 0.831536 | | 1.90E-21 | | 90.4216 | 0.0003 | 265627 |
| rs6870556 | 5 | 31134837 | | A/G | | -0.0185632 | 0.00264149 | | 0.624366 | | 2.10E-12 | | 49.3861 | 0.0002 | 265627 |
| rs6882422 | 5 | 135430668 | | A/G | | -0.0313659 | 0.00400607 | | 0.116759 | | 4.90E-15 | | 61.3021 | 0.0002 | 265627 |
| rs6960700 | 7 | 96146489 | | C/T | | 0.0452441 | 0.002696 | | 0.662211 | | 3.30E-63 | | 281.6313 | 0.0011 | 265627 |
| rs6976382 | 7 | 83637487 | | C/G | | 0.0199436 | 0.00334155 | | 0.177207 | | 2.40E-09 | | 35.6211 | 0.0001 | 265627 |
| rs6977448 | 7 | 132324508 | | T/A | | 0.0199132 | 0.00357123 | | 0.149061 | | 2.50E-08 | | 31.0916 | 0.0001 | 265627 |
| rs6977460 | 7 | 138667788 | | G/A | | -0.016514 | 0.00290829 | | 0.258711 | | 1.40E-08 | | 32.2423 | 0.0001 | 265627 |
| rs6985162 | 8 | 109009951 | | C/A | | -0.0151553 | 0.00270643 | | 0.359088 | | 2.10E-08 | | 31.3568 | 0.0001 | 265627 |
| rs6999846 | 8 | 80813426 | | C/T | | -0.0215044 | 0.00269998 | | 0.654836 | | 1.70E-15 | | 63.4352 | 0.0002 | 265627 |
| rs7014448 | 8 | 127990833 | | G/T | | -0.0237662 | 0.00274361 | | 0.681059 | | 4.60E-18 | | 75.0363 | 0.0003 | 265627 |
| rs7040344 | 9 | 133452158 | | T/C | | -0.0278068 | 0.00269742 | | 0.350974 | | 6.40E-25 | | 106.2678 | 0.0004 | 265627 |
| rs7072196 | 10 | 3581221 | | G/T | | 0.0169672 | 0.00261496 | | 0.597851 | | 8.70E-11 | | 42.1005 | 0.0002 | 265627 |
| rs7090401 | 10 | 115107328 | | T/C | | -0.0222391 | 0.00284341 | | 0.718309 | | 5.20E-15 | | 61.1719 | 0.0002 | 265627 |
| rs7102 | 16 | 11642242 | | C/T | | -0.0198525 | 0.00266404 | | 0.365501 | | 9.20E-14 | | 55.5323 | 0.0002 | 265627 |
| rs7121746 | 11 | 112437007 | | G/A | | -0.0552692 | 0.00260745 | | 0.586691 | | 1.00E-99 | | 449.2945 | 0.0017 | 265627 |
| rs7126943 | 11 | 27303937 | | T/C | | -0.0445089 | 0.00265881 | | 0.635615 | | 6.70E-63 | | 280.2308 | 0.0011 | 265627 |
| rs71338573 | 20 | 6752183 | | T/C | | 0.0400528 | 0.00493329 | | 0.072735 | | 4.70E-16 | | 65.9158 | 0.0002 | 265627 |
| rs71378512 | 16 | 410178 | | A/G | | -0.0926027 | 0.00669804 | | 0.047206 | | 1.80E-43 | | 191.1387 | 0.0007 | 265627 |
| rs71390846 | 16 | 86714715 | | C/G | | -0.0421586 | 0.00328775 | | 0.188706 | | 1.20E-37 | | 164.4264 | 0.0006 | 265627 |
| rs71454826 | 12 | 1639084 | | T/G | | 0.038756 | 0.00297498 | | 0.246276 | | 8.60E-39 | | 169.7096 | 0.0006 | 265627 |
| rs7167692 | 15 | 85660184 | | C/T | | -0.041836 | 0.00576018 | | 0.052165 | | 3.80E-13 | | 52.7503 | 0.0002 | 265627 |
| rs7212160 | 17 | 29836853 | | A/G | | -0.0195623 | 0.00295899 | | 0.742982 | | 3.80E-11 | | 43.7069 | 0.0002 | 265627 |
| rs7235010 | 18 | 20724810 | | A/G | | -0.0194138 | 0.00311418 | | 0.784737 | | 4.50E-10 | | 38.8625 | 0.0001 | 265627 |
| rs7255601 | 19 | 33549775 | | C/G | | 0.0966584 | 0.00440144 | | 0.093591 | | 6.90E-107 | | 482.2657 | 0.0018 | 265627 |
| rs72805220 | 16 | 51903981 | | A/C | | -0.0671715 | 0.00516994 | | 0.067691 | | 1.30E-38 | | 168.8091 | 0.0006 | 265627 |
| rs72868882 | 6 | 55644601 | | T/G | | 0.0488871 | 0.00526465 | | 0.063618 | | 1.60E-20 | | 86.2276 | 0.0003 | 265627 |
| rs72945659 | 6 | 107931983 | | T/C | | 0.0246608 | 0.00403958 | | 0.112825 | | 1.00E-09 | | 37.2682 | 0.0001 | 265627 |
| rs73029259 | 6 | 164111348 | | A/T | | -0.0331283 | 0.00378899 | | 0.130108 | | 2.30E-18 | | 76.4448 | 0.0003 | 265627 |
| rs73238180 | 3 | 142123392 | | A/G | | -0.0239849 | 0.00428132 | | 0.099813 | | 2.10E-08 | | 31.3846 | 0.0001 | 265627 |
| rs7324365 | 13 | 100601327 | | C/T | | -0.0228988 | 0.00322925 | | 0.802284 | | 1.30E-12 | | 50.2827 | 0.0002 | 265627 |
| rs7329483 | 13 | 24242801 | | G/A | | -0.0222915 | 0.00397701 | | 0.881143 | | 2.10E-08 | | 31.4168 | 0.0001 | 265627 |
| rs734703 | 8 | 6388155 | | G/A | | 0.0180732 | 0.00284418 | | 0.719612 | | 2.10E-10 | | 40.3787 | 0.0002 | 265627 |
| rs73520180 | 9 | 118756021 | | C/T | | 0.0305576 | 0.00480238 | | 0.077168 | | 2.00E-10 | | 40.4876 | 0.0002 | 265627 |
| rs74119759 | 10 | 20143109 | | T/C | | -0.027414 | 0.00351994 | | 0.15944 | | 6.80E-15 | | 60.6556 | 0.0002 | 265627 |
| rs74252295 | 17 | 7785905 | | G/A | | -0.0398997 | 0.00485356 | | 0.075588 | | 2.00E-16 | | 67.5795 | 0.0003 | 265627 |
| rs745429 | 5 | 36649049 | | A/G | | 0.0244886 | 0.00361086 | | 0.149736 | | 1.20E-11 | | 45.9942 | 0.0002 | 265627 |
| rs74543960 | 12 | 124501208 | | G/A | | 0.0246354 | 0.00380103 | | 0.132437 | | 9.10E-11 | | 42.0062 | 0.0002 | 265627 |
| rs751979 | 16 | 51169234 | | G/C | | 0.022704 | 0.00268288 | | 0.350134 | | 2.60E-17 | | 71.6142 | 0.0003 | 265627 |
| rs7520984 | 1 | 200710318 | | C/G | | -0.0180232 | 0.00287373 | | 0.731707 | | 3.60E-10 | | 39.334 | 0.0001 | 265627 |
| rs75230517 | 6 | 45106438 | | C/G | | -0.0999014 | 0.00580265 | | 0.05095 | | 2.00E-66 | | 296.4061 | 0.0011 | 265627 |
| rs75663481 | 14 | 75787011 | | G/A | | 0.0220683 | 0.00291687 | | 0.287967 | | 3.90E-14 | | 57.2401 | 0.0002 | 265627 |
| rs7577950 | 2 | 70795622 | | T/C | | 0.0149768 | 0.00259148 | | 0.579248 | | 7.50E-09 | | 33.3994 | 0.0001 | 265627 |
| rs7583768 | 2 | 238416786 | | G/A | | -0.0233563 | 0.00301987 | | 0.235011 | | 1.00E-14 | | 59.8175 | 0.0002 | 265627 |
| rs7584373 | 2 | 68079211 | | A/G | | -0.0173474 | 0.00268093 | | 0.344669 | | 9.80E-11 | | 41.8692 | 0.0002 | 265627 |
| rs7585120 | 2 | 118840782 | | T/C | | 0.017025 | 0.00290014 | | 0.266608 | | 4.30E-09 | | 34.4614 | 0.0001 | 265627 |
| rs75977651 | 1 | 219794385 | | T/C | | -0.0357016 | 0.0042546 | | 0.101316 | | 4.80E-17 | | 70.4133 | 0.0003 | 265627 |
| rs7603114 | 2 | 40666351 | | G/A | | -0.0228505 | 0.00256853 | | 0.483744 | | 5.80E-19 | | 79.1442 | 0.0003 | 265627 |
| rs7633119 | 3 | 30736573 | | C/T | | -0.0169722 | 0.00271663 | | 0.332455 | | 4.20E-10 | | 39.0312 | 0.0001 | 265627 |
| rs7636668 | 3 | 56278593 | | T/C | | 0.020999 | 0.00257468 | | 0.528806 | | 3.50E-16 | | 66.5193 | 0.0003 | 265627 |
| rs76604417 | 1 | 119425396 | | C/T | | 0.0293884 | 0.00387918 | | 0.123705 | | 3.60E-14 | | 57.3943 | 0.0002 | 265627 |
| rs7699480 | 4 | 145607846 | | T/C | | -0.0195818 | 0.00257298 | | 0.56138 | | 2.70E-14 | | 57.9201 | 0.0002 | 265627 |
| rs7703751 | 5 | 122831981 | | T/A | | -0.0240449 | 0.00292669 | | 0.25693 | | 2.10E-16 | | 67.4977 | 0.0003 | 265627 |
| rs7720275 | 5 | 158246650 | | C/T | | -0.0258304 | 0.00340194 | | 0.169952 | | 3.10E-14 | | 57.6508 | 0.0002 | 265627 |
| rs7732038 | 5 | 172998191 | | C/T | | 0.020138 | 0.00260947 | | 0.602632 | | 1.20E-14 | | 59.5559 | 0.0002 | 265627 |
| rs7741021 | 6 | 127468274 | | C/A | | 0.0755656 | 0.00255153 | | 0.478075 | | 9.40E-193 | | 877.089 | 0.0033 | 265627 |
| rs77431781 | 4 | 124534992 | | T/A | | -0.0284472 | 0.00494622 | | 0.076036 | | 8.90E-09 | | 33.0772 | 0.0001 | 265627 |
| rs77847666 | 7 | 120501308 | | C/T | | 0.0752347 | 0.0066364 | | 0.03823 | | 8.60E-30 | | 128.5192 | 0.0005 | 265627 |
| rs78058190 | 2 | 219699999 | | A/G | | 0.0389455 | 0.00655393 | | 0.050583 | | 2.80E-09 | | 35.3108 | 0.0001 | 265627 |
| rs78161283 | 4 | 119792345 | | T/C | | -0.0345236 | 0.00563518 | | 0.055679 | | 9.00E-10 | | 37.533 | 0.0001 | 265627 |
| rs78416576 | 1 | 22543368 | | T/C | | -0.0460342 | 0.00398398 | | 0.118993 | | 7.00E-31 | | 133.513 | 0.0005 | 265627 |
| rs78432519 | 6 | 21891576 | | T/C | | -0.0389176 | 0.00402298 | | 0.112635 | | 3.90E-22 | | 93.5822 | 0.0004 | 265627 |
| rs78556801 | 16 | 23149089 | | T/G | | -0.0187654 | 0.0027846 | | 0.305242 | | 1.60E-11 | | 45.4137 | 0.0002 | 265627 |
| rs78667121 | 13 | 43200103 | | A/G | | 0.0531217 | 0.00748676 | | 0.030058 | | 1.30E-12 | | 50.3446 | 0.0002 | 265627 |
| rs7870885 | 9 | 89719679 | | G/A | | 0.0174889 | 0.00276418 | | 0.315701 | | 2.50E-10 | | 40.0303 | 0.0002 | 265627 |
| rs7902212 | 10 | 20382142 | | C/T | | 0.0186683 | 0.00256584 | | 0.539998 | | 3.40E-13 | | 52.9355 | 0.0002 | 265627 |
| rs7919753 | 10 | 54439699 | | G/A | | -0.125871 | 0.00409953 | | 0.10931 | | 1.00E-200 | | 942.714 | 0.0035 | 265627 |
| rs7961324 | 12 | 78220653 | | G/A | | -0.0147235 | 0.00269491 | | 0.343314 | | 4.70E-08 | | 29.849 | 0.0001 | 265627 |
| rs79640667 | 12 | 49358392 | | C/T | | 0.0289464 | 0.00503076 | | 0.069164 | | 8.70E-09 | | 33.1069 | 0.0001 | 265627 |
| rs79854277 | 20 | 29844516 | | T/C | | 0.0324678 | 0.00581268 | | 0.052523 | | 2.30E-08 | | 31.1996 | 0.0001 | 265627 |
| rs8002850 | 13 | 22811940 | | A/G | | -0.0283093 | 0.00271357 | | 0.334066 | | 1.80E-25 | | 108.8361 | 0.0004 | 265627 |
| rs8068674 | 17 | 76907445 | | T/C | | -0.015854 | 0.00260669 | | 0.413033 | | 1.20E-09 | | 36.991 | 0.0001 | 265627 |
| rs8134775 | 21 | 35591826 | | C/T | | 0.0241235 | 0.00321432 | | 0.199751 | | 6.10E-14 | | 56.3247 | 0.0002 | 265627 |
| rs847148 | 2 | 176970456 | | T/A | | -0.0172654 | 0.00272256 | | 0.326719 | | 2.30E-10 | | 40.2157 | 0.0002 | 265627 |
| rs848188 | 1 | 16277082 | | C/A | | 0.0380159 | 0.00422053 | | 0.101627 | | 2.10E-19 | | 81.1322 | 0.0003 | 265627 |
| rs868127 | 15 | 70402095 | | T/C | | -0.0200228 | 0.00287467 | | 0.285417 | | 3.30E-12 | | 48.5144 | 0.0002 | 265627 |
| rs884205 | 18 | 60054857 | | C/A | | 0.0231676 | 0.00297106 | | 0.747708 | | 6.30E-15 | | 60.8045 | 0.0002 | 265627 |
| rs912146 | 13 | 24828827 | | A/G | | 0.0236688 | 0.00351585 | | 0.156762 | | 1.70E-11 | | 45.3199 | 0.0002 | 265627 |
| rs9257703 | 6 | 29278505 | | G/T | | -0.0205029 | 0.00293137 | | 0.747215 | | 2.70E-12 | | 48.92 | 0.0002 | 265627 |
| rs9290351 | 3 | 168692352 | | T/G | | 0.0230388 | 0.00390703 | | 0.121783 | | 3.70E-09 | | 34.7715 | 0.0001 | 265627 |
| rs9324144 | 1 | 85332649 | | T/C | | -0.0206334 | 0.00283258 | | 0.702466 | | 3.20E-13 | | 53.0608 | 0.0002 | 265627 |
| rs9326769 | 5 | 108653720 | | T/C | | -0.0172331 | 0.00260051 | | 0.412309 | | 3.40E-11 | | 43.9144 | 0.0002 | 265627 |
| rs9340903 | 6 | 152266043 | | T/C | | -0.0554448 | 0.00744449 | | 0.030387 | | 9.50E-14 | | 55.4688 | 0.0002 | 265627 |
| rs938662 | 2 | 45864578 | | G/A | | 0.0205446 | 0.00283962 | | 0.710026 | | 4.70E-13 | | 52.3446 | 0.0002 | 265627 |
| rs947091 | 10 | 31054186 | | A/G | | 0.0382616 | 0.00256866 | | 0.482882 | | 3.50E-50 | | 221.8757 | 0.0008 | 265627 |
| rs9474632 | 6 | 53578160 | | A/G | | -0.015074 | 0.00267475 | | 0.360122 | | 1.70E-08 | | 31.7605 | 0.0001 | 265627 |
| rs9521510 | 13 | 110426871 | | C/T | | -0.0180526 | 0.00274749 | | 0.322891 | | 5.00E-11 | | 43.1722 | 0.0002 | 265627 |
| rs9594738 | 13 | 42952145 | | T/C | | -0.0501234 | 0.00255951 | | 0.487302 | | 2.10E-85 | | 383.4989 | 0.0014 | 265627 |
| rs9606139 | 22 | 19679303 | | A/G | | -0.109218 | 0.0042032 | | 0.108311 | | 7.40E-149 | | 675.1885 | 0.0025 | 265627 |
| rs9631060 | 2 | 54369588 | | G/A | | -0.0217054 | 0.00308878 | | 0.21905 | | 2.10E-12 | | 49.3808 | 0.0002 | 265627 |
| rs9896306 | 17 | 63278038 | | A/C | | -0.0234836 | 0.0028786 | | 0.272934 | | 3.40E-16 | | 66.5524 | 0.0003 | 265627 |
| rs9990527 | 4 | 1324360 | | T/C | | -0.0326066 | 0.00335086 | | 0.177031 | | 2.20E-22 | | 94.6881 | 0.0004 | 265627 |
| **Genetic variants of Lumbar spine bone mineral density** | | | | | | | | | | | |  |  |  |  |
| rs1023940 | 6 | 151932778 | | G/A | | -0.064541 | 0.00876 | | 0.534845 | | 6.47E-13 | | 54.2805 | 0.0012 | 44731 |
| rs11002249 | 10 | 79447228 | | T/C | | 0.069743 | 0.011142 | | 0.235402 | | 1.01E-09 | | 39.1792 | 0.0009 | 44731 |
| rs11024028 | 11 | 16756873 | | G/C | | 0.065776 | 0.011325 | | 0.147176 | | 1.44E-08 | | 33.7318 | 0.0008 | 44731 |
| rs11680288 | 2 | 166603281 | | A/G | | -0.054235 | 0.008933 | | 0.428958 | | 3.12E-09 | | 36.8591 | 0.0008 | 44731 |
| rs11692564 | 2 | 119545994 | | T/C | | 0.23782 | 0.039471 | | 0.0127287 | | 4.10E-09 | | 36.3012 | 0.0008 | 44731 |
| rs13046645 | 21 | 36818136 | | A/T | | -0.055863 | 0.009829 | | 0.276293 | | 2.92E-08 | | 32.3006 | 0.0007 | 44731 |
| rs1357651 | 7 | 38097862 | | G/T | | 0.068117 | 0.009151 | | 0.645187 | | 3.75E-13 | | 55.4057 | 0.0012 | 44731 |
| rs2235811 | 20 | 10644158 | | G/A | | -0.054301 | 0.009045 | | 0.537709 | | 4.66E-09 | | 36.0395 | 0.0008 | 44731 |
| rs2291467 | 11 | 68216756 | | T/C | | -0.077421 | 0.010148 | | 0.228162 | | 9.64E-14 | | 58.2019 | 0.0013 | 44731 |
| rs2566752 | 1 | 68656697 | | C/T | | 0.082922 | 0.008947 | | 0.41074 | | 1.49E-19 | | 85.8945 | 0.0019 | 44731 |
| rs35681117 | 7 | 37959021 | | T/C | | 0.055162 | 0.009645 | | 0.237947 | | 2.39E-08 | | 32.7082 | 0.0007 | 44731 |
| rs6965122 | 7 | 96133319 | | G/A | | -0.061835 | 0.009266 | | 0.265712 | | 7.40E-11 | | 44.5312 | 0.001 | 44731 |
| rs71390846 | 16 | 86714715 | | C/G | | -0.064092 | 0.011373 | | 0.173667 | | 3.80E-08 | | 31.7569 | 0.0007 | 44731 |
| rs73326583 | 14 | 91465976 | | A/C | | 0.072363 | 0.011887 | | 0.160382 | | 2.83E-09 | | 37.0569 | 0.0008 | 44731 |
| rs7524102 | 1 | 22698447 | | G/A | | 0.089822 | 0.011494 | | 0.198329 | | 2.41E-14 | | 61.0666 | 0.0014 | 44731 |
| rs7807953 | 7 | 121000718 | | T/C | | 0.075102 | 0.009698 | | 0.263325 | | 4.11E-14 | | 59.968 | 0.0013 | 44731 |
| rs78667121 | 13 | 43200103 | | A/G | | 0.149862 | 0.025668 | | 0.0221957 | | 1.21E-08 | | 34.0863 | 0.0008 | 44731 |
| rs884205 | 18 | 60054857 | | C/A | | 0.062204 | 0.010212 | | 0.786794 | | 2.77E-09 | | 37.1019 | 0.0008 | 44731 |
| rs894738 | 12 | 54417525 | | A/G | | -0.062783 | 0.009136 | | 0.333492 | | 2.00E-11 | | 47.2229 | 0.0011 | 44731 |
| rs9533094 | 13 | 42965837 | | G/A | | -0.082629 | 0.008741 | | 0.404296 | | 2.80E-20 | | 89.3559 | 0.002 | 44731 |
| rs9749364 | 19 | 33538783 | | G/T | | 0.11438 | 0.018078 | | 0.133492 | | 6.64E-10 | | 40.0295 | 0.0009 | 44731 |
| rs9921222 | 16 | 375782 | | T/C | | -0.053345 | 0.008789 | | 0.461177 | | 3.16E-09 | | 36.8374 | 0.0008 | 44731 |

Supplementary Table 4. Instrument variants for the Mendelian randomization analyses of IVDD on OP and BMD

| **SNP** | **Chr** | **Pos** | **EA/OA** | **Beta** | **SE** | **MAF** | **P** | **F** | **R2** | **Sample Size** |
| --- | --- | --- | --- | --- | --- | --- | --- | --- | --- | --- |
| rs3010043 | 1 | 183942175 | G/A | -0.084 | 0.014 | 0.791 | 4.73E-09 | 34.1771 | 0.0002 | 184683 |
| rs4473430 | 2 | 69582895 | T/C | -0.065 | 0.012 | 0.552 | 2.42E-08 | 30.9589 | 0.0002 | 184683 |
| rs3135840 | 4 | 1796539 | T/A | -0.082 | 0.013 | 0.263 | 9.27E-10 | 37.6420 | 0.0002 | 184683 |
| rs6470763^a^ | 8 | 130720646 | C/G | -0.092 | 0.016 | 0.165 | 6.82E-09 | 33.4793 | 0.0002 | 184683 |
| rs4284332 | 10 | 73733952 | C/T | 0.065 | 0.012 | 0.557 | 3.47E-08 | 30.5796 | 0.0002 | 184683 |
| rs17487277 | 18 | 50718944 | G/C | 0.079 | 0.012 | 0.404 | 3.42E-11 | 44.2558 | 0.0002 | 184683 |

^a^ ^rs6470763 is directly associated with heel BMD (Beta= 0.021, p= 7.60×10-11), which is not accord with the third assumption of MR analysis. Hence, we deleted it for the analysis of IVDD and the risk of eBMD.^

Supplementary Table 5. Statistical power for the Mendelian randomization analyses of BMD on IVDD

| **Exposure** | **Outcome** | **Variance** | **Sample size of outcome** | **Type-I error rate** | **Proportion of cases** | **Selected scenarios** | | | |
| --- | --- | --- | --- | --- | --- | --- | --- | --- | --- |
|  |  |  |  |  |  | **OR =0.9** | **OR =1.0** | **OR =1.1** | **OR =1.2** |
| **OP** | **IVDD** | 0.0002 | 184683 | **0.05** | 0.108299 | 0.50 | 0.05 | 0.48 | 0.97 |
| **TB-BMD** | **IVDD** | 0.0905 | 184683 | **0.05** | 0.108299 | 0.98 | 0.05 | 0.97 | 1.00 |
| **FN-BMD** | **IVDD** | 0.021 | 184683 | **0.05** | 0.108299 | 0.65 | 0.05 | 0.63 | 1.00 |
| **FA-BMD** | **IVDD** | 0.0165 | 184683 | **0.05** | 0.108299 | 0.50 | 0.05 | 0.49 | 0.97 |
| **LS-BMD** | **IVDD** | 0.0227 | 184683 | **0.05** | 0.108299 | 0.77 | 0.05 | 0.75 | 1.00 |
| **eBMD** | **IVDD** | 0.1427 | 184683 | **0.05** | 0.108299 | 1.0 | 0.05 | 1.00 | 1.00 |

IVDD, Intervertebral disc degeneration; TB-BMD, Total body bone mineral density; FN-BMD, Femoral neck bone mineral density; FA-BMD, Forearm bone mineral density; LS-BMD; eBMD, Heel bone mineral density;


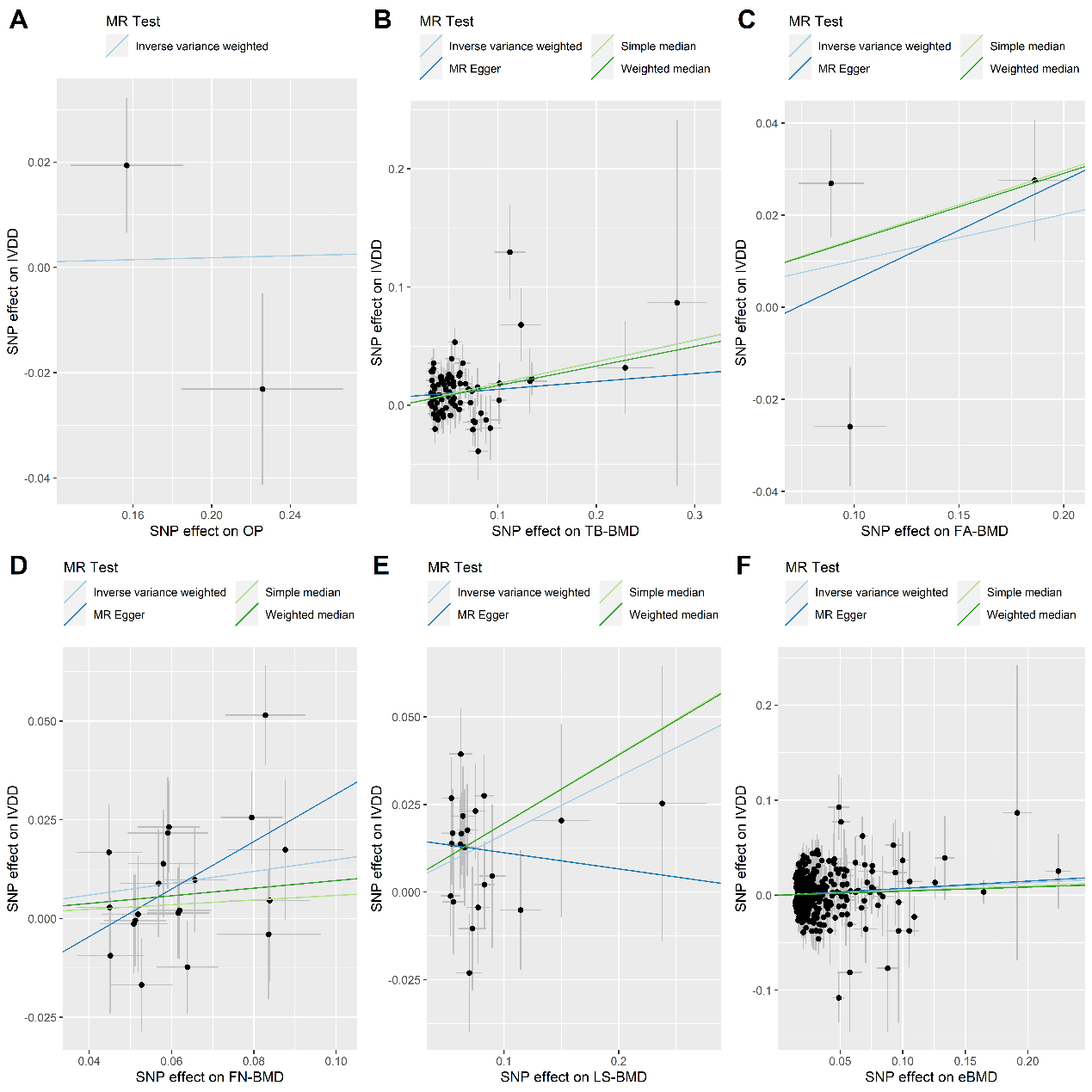


Supplementary Figure 1. Scatter plot of the relationship of OP and IVDD using inverse-variance weighted, simple median, MR-Egger, and weighted median.

IVDD – Intervertebral disc degeneration; OP – Osteoporosis; TB-BMD – Total body bone mineral density; FN-BMD – Femoral neck bone mineral density; FA-BMD – Forearm bone mineral density; LS-BMD – Lumbar spine bone mineral density; eBMD – heel bone mineral density; IVW – Inverse variance weighted.


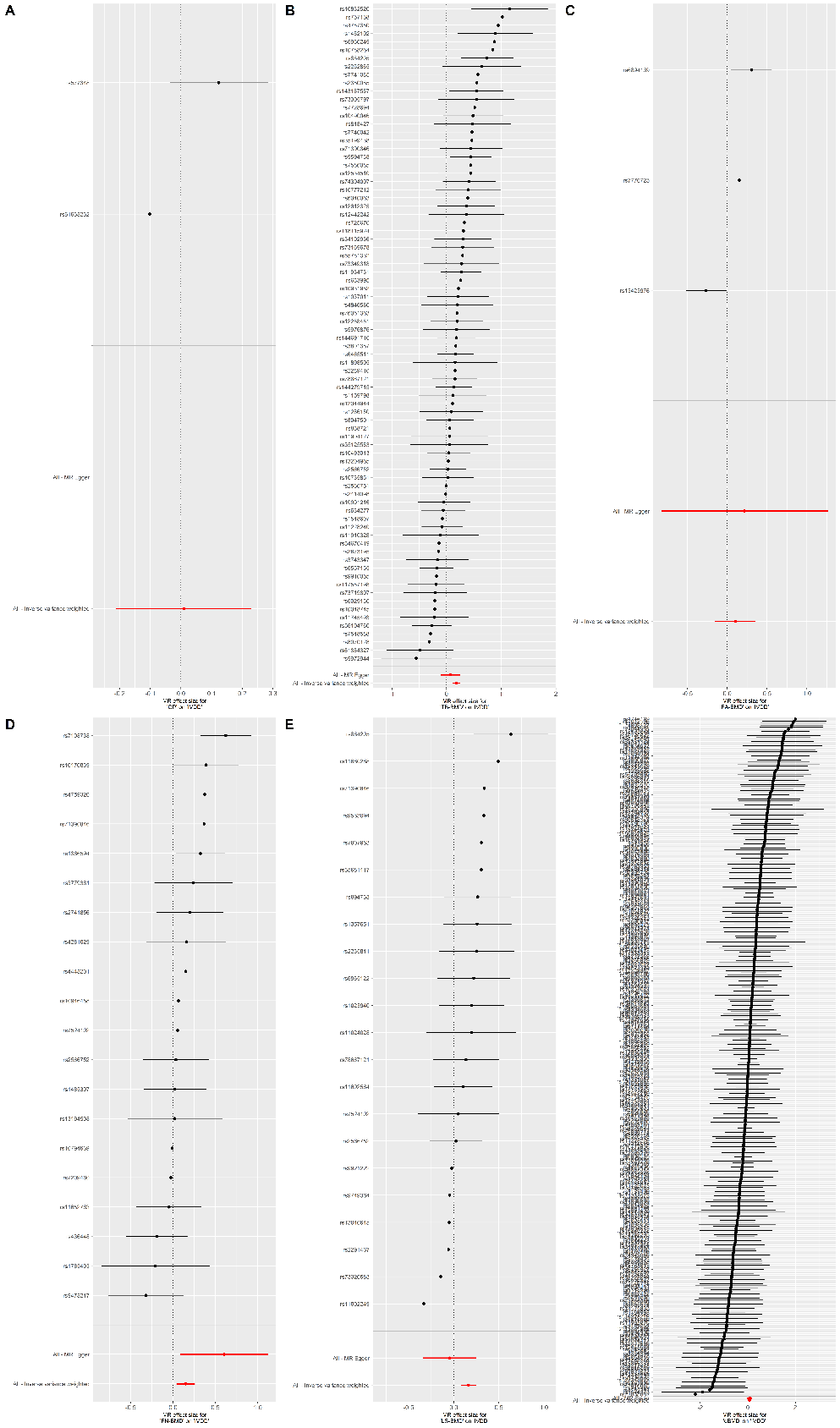


Supplementary Figure 2. Forest plot for causal effect of OP (A), TB-BMD (B), FN-BMD (C), FA-BMD (D), LS-BMD (E), eBMD (F) on IVDD.


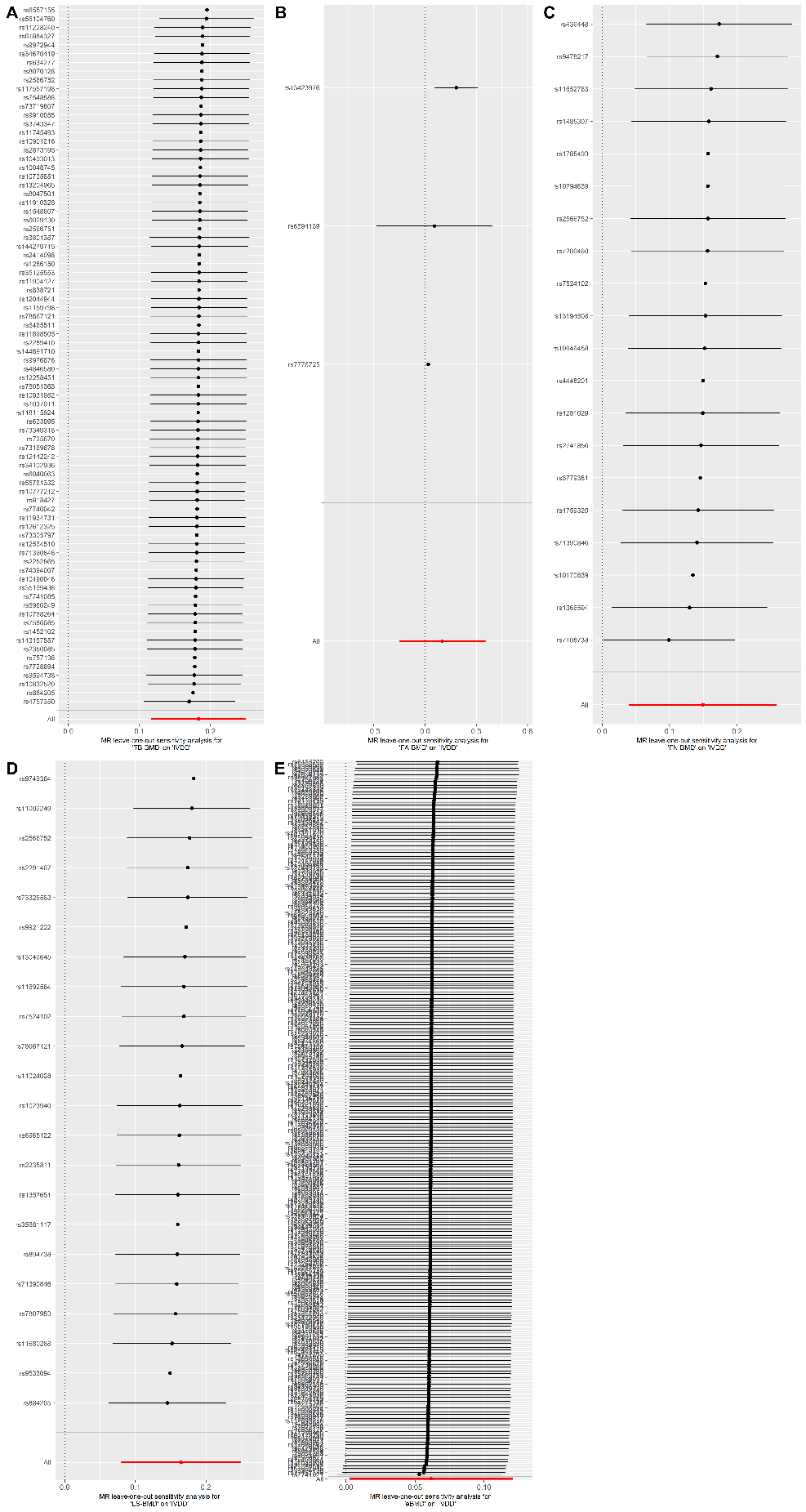


Supplementary Figure 3. Leave-one-out plot for causal effect of TB-BMD (A), FN-BMD (B), FA-BMD (C), LS-BMD (D), eBMD (E) on IVDD.


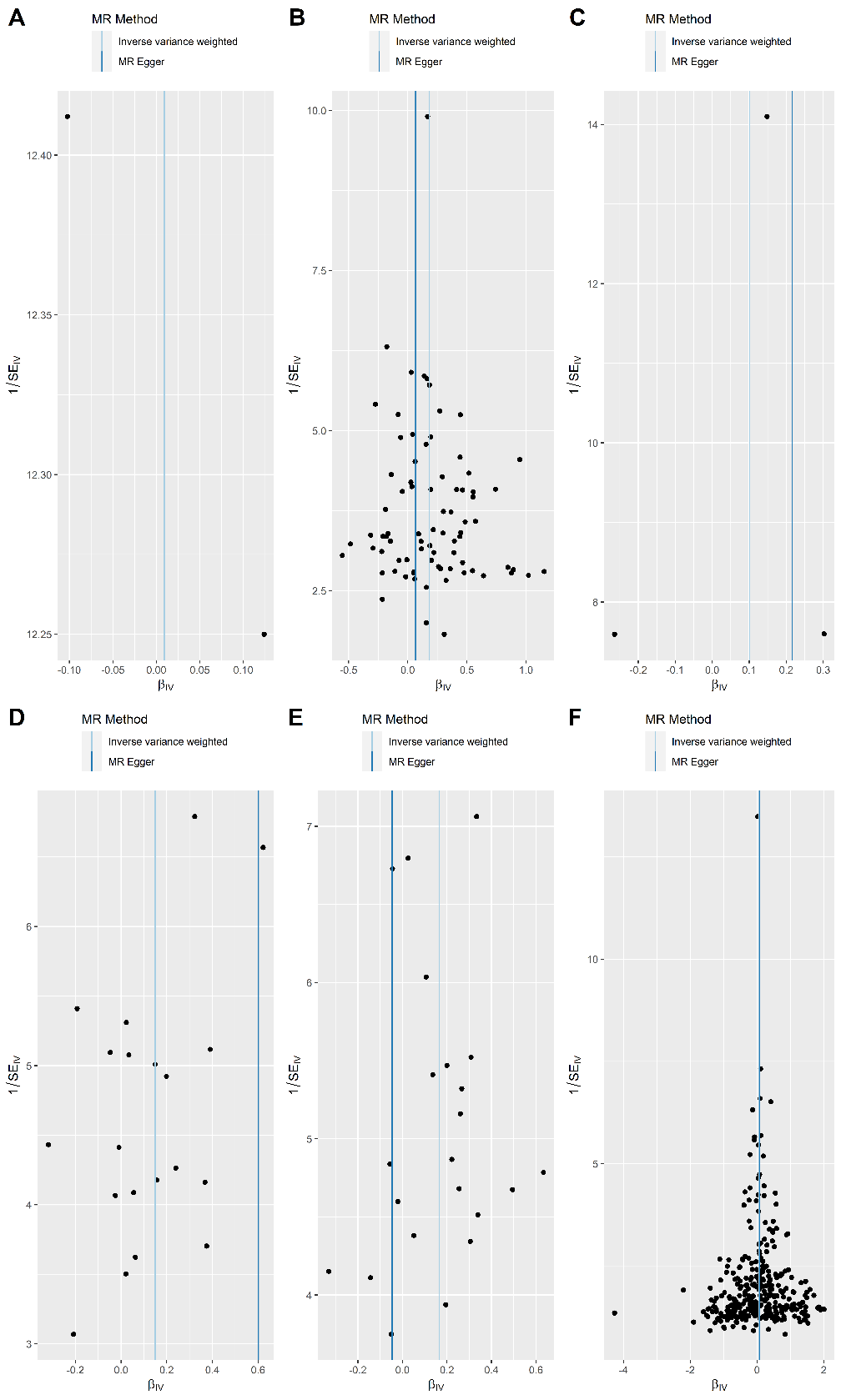


Supplementary Figure 4. Funnel plot for causal effect of OP (A), TB-BMD (B), FN-BMD (C), FA-BMD (D), LS-BMD (E), and eBMD (F) on IVDD.

IVDD – Intervertebral disc degeneration; OP – Osteoporosis; TB-BMD – Total body bone mineral density; FN-BMD – Femoral neck bone mineral density; FA-BMD – Forearm bone mineral density; LS-BMD – Lumbar spine bone mineral density; eBMD – heel bone mineral density.
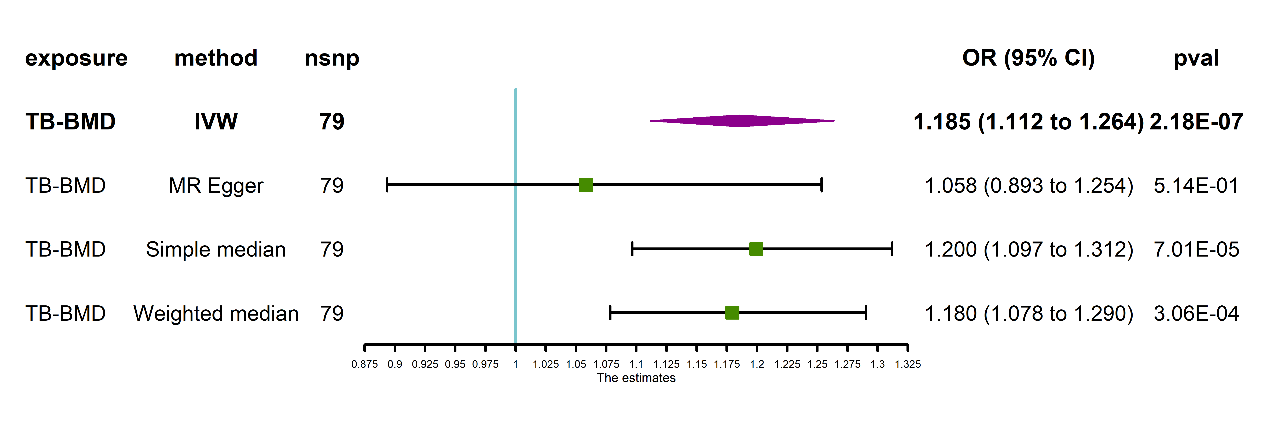


Supplementary Figure 5. Mendelian randomization analysis results for the effects of TB-BMD on IVDD after removal of potentially pleiotropic SNP (rs4846580).

IVDD – Intervertebral disc degeneration; TB-BMD – Total body bone mineral density; IVW – Inverse variance weighted; nsnp – number of single nucleotide polymorphisms; CI – Confidence interval.


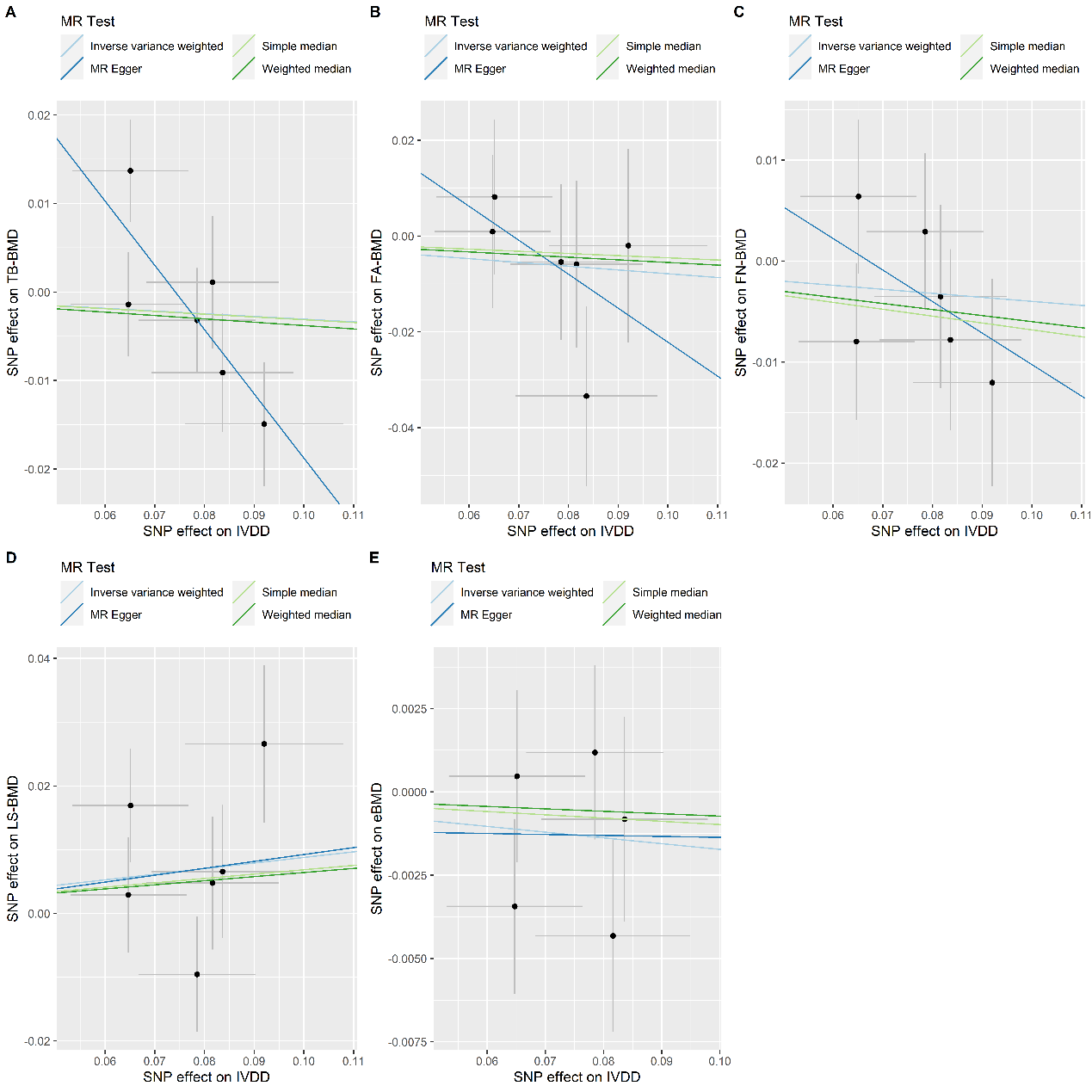


Supplementary Figure 6. Scatter plot of the causal effect of IVDD and BMD at different sites using inverse-variance weighted, simple median, MR-Egger, and weighted median. (A) Causal estimates for IVDD on TB-BMD. (B) Causal estimates for IVDD on FA-BMD. (C) Causal estimates for IVDD on FN-BMD. (D) Causal estimates for IVDD on LS-BMD. (E) Causal estimates for IVDD on eBMD. IVDD – Intervertebral disc degeneration; OP – Osteoporosis; TB-BMD – Total body bone mineral density; FN-BMD – Femoral neck bone mineral density; FA-BMD – Forearm bone mineral density; LS-BMD – Lumbar spine bone mineral density; eBMD – heel bone mineral density; IVW – Inverse variance weighted.


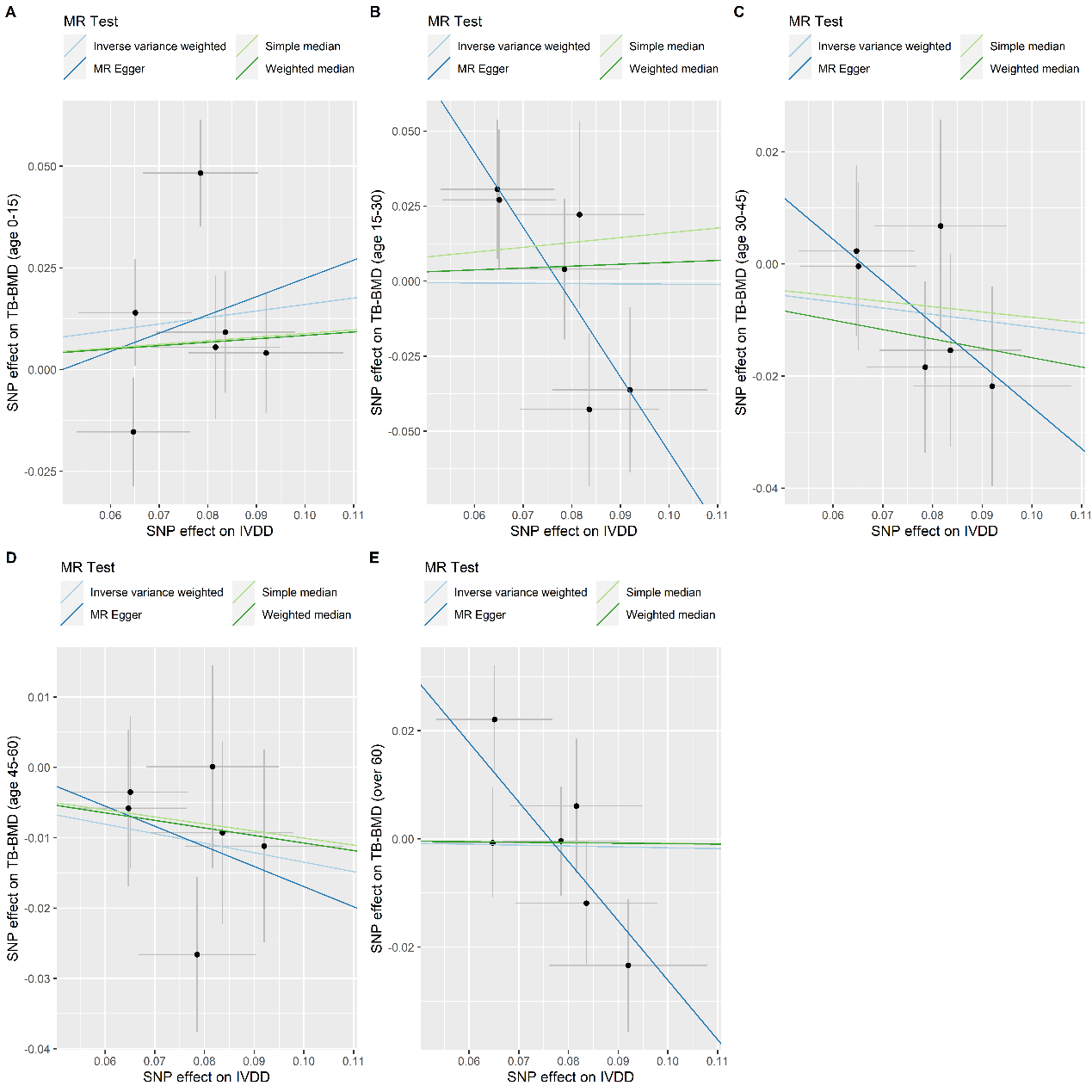


Supplementary Figure 7. Scatter plot of the causal effect of IVDD and BMD in different age groups using inverse-variance weighted, simple median, MR-Egger, and weighted median. (A) Causal estimates for IVDD on TB-BMD (age 0-15). (B) Causal estimates for IVDD on TB-BMD (age 15-30). (C) Causal estimates for IVDD on TB-BMD (age 30-45). (D) Causal estimates for AS on TB-BMD (age 45-60). (E) Causal estimates for IVDD on TB-BMD (age over 60). IVDD – Intervertebral disc degeneration; OP – Osteoporosis; TB-BMD – Total body bone mineral density; IVW – Inverse variance weighted.


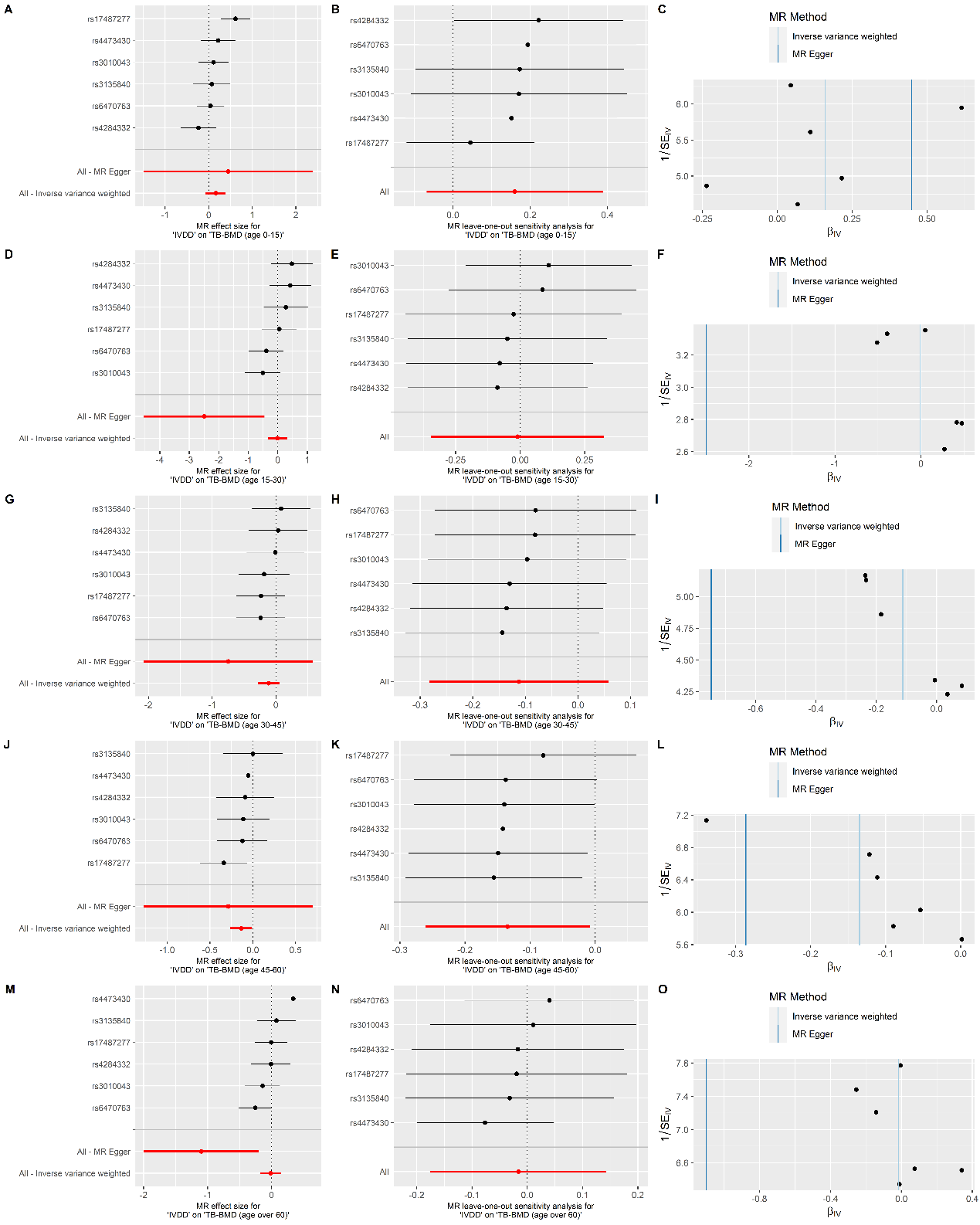


Supplementary Figure 8. MR sensitivity analysis of IVDD on BMD at different ages. TB-BMD – Total body bone mineral density.


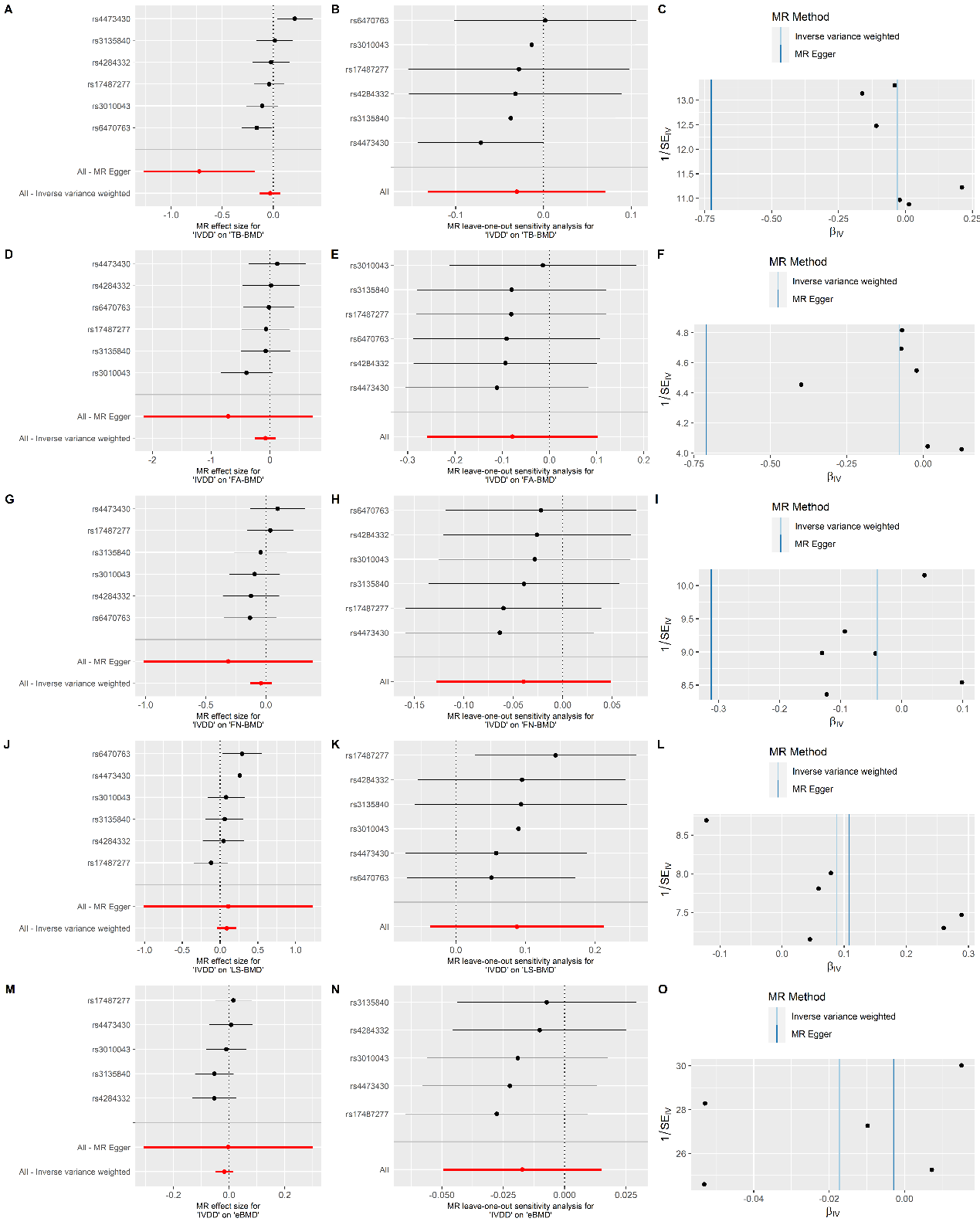
Supplementary Figure 9. MR sensitivity analysis of IVDD on BMD at different sites.

IVDD – Intervertebral disc degeneration; OP – Osteoporosis; TB-BMD – Total body bone mineral density; FN-BMD – Femoral neck bone mineral density; FA-BMD – Forearm bone mineral density; LS-BMD – Lumbar spine bone mineral density; eBMD – heel bone mineral density.
